# Evolution and function of genetic polymorphisms in the *Helicobacter pylori* type IV secretion pilus protein CagL in gastric carcinogenesis

**DOI:** 10.1186/s12964-026-03334-9

**Published:** 2026-10-30

**Authors:** Nicole Tegtmeyer, Judith Romero-Gallo, Bodo Linz, Maria Blanca Piazuelo, Omar Noman, Manfred Rohde, Heinrich Sticht, Richard M. Peek, Steffen Backert

**Affiliations:** 1 Department of Biology, Division of Microbiology, Friedrich-Alexander-Universität Erlangen-Nürnberg, Erlangen, 91058 Germany; 2 Department of Medicine, Vanderbilt University Medical Center, Nashville, TN USA; 3 Central Facility for Microscopy, Helmholtz Centre for Infection Research, Braunschweig, Germany; 4 Division of Bioinformatics, Institute of Biochemistry, Friedrich-Alexander-Universität Erlangen-Nürnberg, 91054 Erlangen, Germany

**Keywords:** Beta-catenin, CagL, DNA damage, DSBs, Helicobacter, Signaling, SNP, T4SS

## Abstract

**Background:**

The cytotoxin-associated genes (*cag*) type IV secretion system (T4SS) of the gastric pathogen *Helicobacter pylori* constitutes a key determinant in gastric cancer (GC) development. The CagL protein represents a VirB5 ortholog that acts as a signalling factor at the surface of the T4SS pilus, which facilitates binding to host target cells to inject T4SS substrates such as the CagA oncoprotein and ADP-heptose. Previous studies reported the presence of a hypervariable region in CagL at amino acid positions 58 and 59 with a proposed role in GC progression, however, studies using a large number of *H. pylori* strains as well as detailed functional experiments are yet missing.

**Methods:**

Here, we investigated the CagL status in 2,267 worldwide *H. pylori* genomes from an evolutionary point of view, concerning gastric disease status, and directly compared the Y58/E59 versus N58/K59 polymorphism in CagL in the GC-associated *H. pylori* strain 7.13 with regard to T4SS-pilus formation and cancer signalling during infection of gastric epithelial cells in vitro and infection of Mongolian gerbils in vivo.

**Results:**

Structural analysis showed that the genetic polymorphism, located in an amino acid loop flanked by two α-helices, affects CagL conformation and functional activity. Remarkably, both strains revealed similar robust T4SS-pilus formation, but functional differences. We demonstrate that wild-type *H. pylori* 7.13 encoding N58/K59 CagL triggered GC development in Mongolian gerbils and characteristic GC signalling events in gastric epithelial cells, including CagA phosphorylation, activation of MAP kinases (p38, ERK1/2 and JNK), β-catenin and cell motility as well as pronounced induction of pro-inflammatory transcription factor NF-κB-dependent interleukin-8 (IL-8) production and DNA double-strand breaks (DSBs), whereas *H. pylori* encoding Y58/E59 CagL elicited markedly attenuated responses.

**Conclusions:**

These data suggest that the Y58/E59 mutation impairs translocation of CagA into host cells, but retains T4SS-pilus formation and ADP-heptose-dependent NF-κB activation. This implies that CagL mutation may act as molecular regulator of the T4SS for “finetuning” CagA delivery, host inflammation and other oncogenic responses. Interestingly, this scenario is similar to previously reported CagY, another T4SS-pilus protein, which can cause the gain or loss of IL-8 production by genetic polymorphisms that occur through recombination events.

**Supplementary Information:**

The online version contains supplementary material available at https://doi.org/10.1186/s12964-026-03334-9.

## Background

*Helicobacter pylori* is a human-specific bacterium that persistently colonizes the stomachs of about half of the human population, and therefore represents one of the most successful pathogens worldwide [1–3]. Genetic studies have demonstrated that the evolutionary origin of *H. pylori* is in Africa [4, 5]. *H. pylori* likely originated from a host jump of *Helicobacter cetorum* colonizing the stomachs of dolphins or whales that happened over 600,000 years ago [6]. Afterwards, *H. pylori* accompanied its new host during the human migrations out-of-Africa about 60,000 years ago, and subsequently spread across all continents. During this long, intimate co-evolution with humans, the bacteria differentiated into discrete geographic *Helicobacter* populations, namely hpAfrica1, hpAfrica2 and hpNEAfrica (in Africa), hpEurope, hpEastAsia and hpAsia2 (in Europe and Asia), hpSahul (in Australia and New Guinea), hpSiberia (in Northern Russia) and hpNorthAsia (previously known as *Helicobacter* subpopulation hspAmerind, in Siberia, and North and South America) [7–9]. While the infection is asymptomatic in most cases, approximately 10–15% of *H. pylori*-positive patients develop serious gastric diseases such as gastric ulcers or gastric cancer (GC). GC represents one of the deadliest human cancers, and is often diagnosed at late stages, which is associated with a poor prognosis for patient survival. Worldwide, GC is the fifth most common form of cancer and the fourth leading cause of cancer-related deaths [3, 10, 11]. The recently announced global GLOBOCAN statistics revealed 660,180 GC-associated deaths and 968,780 new cases of GC in the year 2022 [12]. Notably, the prevalence rates of GC are varying by geographic region with substantially elevated rates in distinct regions of Asia and Eastern Europe [13]. In particular, *H. pylori* infection eventually initiates the well-known Correa cascade, which describes the histopathological progression from gastritis to the intestinal type of GC [14]. Overall, GC development has been recognized as a multifactorial, highly complex and long-term process. *H. pylori*-dependent GC progression is commonly characterized by downregulation of gastric acid secretion, microbial dysbiosis, elevated proliferation of the gastric epithelial cells, DNA damage as well as infiltration of macrophages, polymorphonuclear leukocytes (PMNs) and lymphocytes in the gastric mucosa [1–3, 15–17]. In recent decades, various animal model systems have been developed to study the molecular mechanisms of gastric disease development, most notably the Mongolian gerbils, which allow the functional assessment of individual *H. pylori* virulence genes in the pathogenesis of GC [18, 19].

The disease-associated capacity of *H. pylori* is dependent on the genetic repertoire of individual isolates. Strains that express numerous virulence factors, including the adhesins BabA and HopQ, vacuolating cytotoxin VacA, serine protease HtrA and the cytotoxin-associated genes pathogenicity island (*cag*PAI), trigger pronounced cell-damaging activities and are associated with GC [20–24]. In particular, the *cag*PAI forms a surface-exposed type IV secretion system (T4SS) for the translocation of effector molecules, most notably oncoprotein CagA and adenosine diphosphate-β-d-manno-heptose (ADP-heptose), both of which represent major bacterial drivers of GC development [2, 25]. While intracellular CagA undergoes tyrosine-phosphorylation at the so-called EPIYA sequence motifs and triggers β-catenin-dependent cell proliferation, cell scattering and anti-apoptotic responses [2, 26], ADP-heptose activates the pro-inflammatory transcription factor NF-κB and interleukin-8 (IL-8) expression [27, 28]. The T4SS genes are encoded by the *cag*PAI locus that is about 40 kilobase pairs in size. Depending on the geographical region, the *cag*PAI commonly harbours 27–29 open reading frames, including the *virB1-virB11* orthologous genes that were originally described for the archetypal T4SS machinery from *Agrobacterium tumefaciens* [29–31]. In addition, the *cag*PAI encodes the so-called VirD4 coupling factor, required for CagA translocation, and 16–18 other accessory Cag proteins [32, 33]. The application of cryo-electron tomographic and electron microscopic approaches have unravelled the molecular structures of the Cag T4SS formed in the bacterial membranes and from the purified T4SS core complex, respectively [34–36]. It was proposed that this T4SS core structure is directly connected to an extracellular T4SS-pilus, composed of VirB2 subunits [30, 37]. Immunogold and functional studies have shown that the tip of the T4SS pili contains the VirB5 ortholog CagL [38, 39], which facilitates binding of the host cell receptors integrin-α5β1 [38], integrin-αvβ3 [40], integrin-αvβ5 [41], integrin-αvβ6 [42] and toll-like receptor 5 (TLR5) [43]. Remarkably, mass spectrometry of immunoprecipitation experiments showed that CagL probably acts cooperatively with two other Cag proteins, CagH and CagI, which form a heterotrimer [44]. Each of the three individual genes was essential for both T4SS-mediated CagA delivery and ADP-heptose-dependent IL-8 production [38, 44]. These findings suggested that CagL, CagH and CagI represent crucial functional components of the *H. pylori* T4SS-pilus.

Genetic polymorphisms, including SNPs, in human genes represent a well-known predisposition for GC development [45, 46]. For example, polymorphisms in immunoregulatory genes encoding chemokine IL-1β [47], toll-like receptors [48] as well as E-cadherin (*cdh1*) and homologous recombination enzymes (*ATM*, *BRCA1*, *BRCA2* and *PALB2*) [49] have been significantly associated with GC. GC-specific gene polymorphisms have been also described for *H. pylori*, including the VacA allele s1m1 [50], the CagA phosphorylation motif EPIYA-B [51] and serine protease HtrA alleles S171L [52], D39E and I439V [53]. Genetic polymorphisms have been also reported for the T4SS-pilus protein CagL, in particular, some hypervariable amino acid residues at positions 58 and 59. For instance, in a clinical study from Taiwan, a Y58 and E59 polymorphism was discovered in CagL from 27 GC patients compared to 77 non-GC patients [54, 55]. The authors stated that the Y58/E59 polymorphism represented an up to 4.6-fold increased risk of GC, which was associated with a shift of integrin α5β1 expression and inflammation from the antrum towards the corpus [54]. In a study from India, another CagL polymorphism at the same positions (D58 and K59) was detected in 23 GC patients (3.8-fold elevated) compared to 33 non-ulcer dyspepsia controls [56]. Similar observations were made in Turkey, where the D58/K59 polymorphism was present in 16 GC patients [57]. Contrary, a group from Iran reported a 6.5-fold higher association of the D58 polymorphism in CagL from 21 patients with peptic ulcer disease compared to 3 GC and 4 gastritis patients, while an N58 polymorphism was predominantly observed in 11 GC samples [58]. Another study analysed worldwide CagL sequences from 309 patients with available health status [59]. Markedly, the authors noted another polymorphism (E59/I60) to be overrepresented in 37 GC patients, whereas D58/E62 sequences were underrepresented in this cohort. Taken together, these previous data from numerous research labs in different countries did not provide a consistent picture for the potential role of a particular CagL polymorphism in clinical *H. pylori* strains in relation to specific gastric disease. In addition, extensive statistical analyses in the above studies were severely hampered by the relatively low numbers of analysed patients from specific geographic locations. Moreover, in vitro infection experiments using CagL mutants with respective Y58/E59 or N58/K59 amino acid substitutions in the *H. pylori* gastritis strain 26695 did not provide consistent results with regard to CagA phosphorylation and induction of IL-8 upon infection of AGS gastric epithelial cells [60, 61]. Thus, there is a strong controversy in the literature about the role of specific CagL polymorphisms in the development of GC and other gastric diseases. In the present study, we investigated these CagL polymorphisms in a very large number (2,267) of worldwide *H. pylori* genomes from an evolutionary point of view and in relation to gastric diseases. In addition, we directly compared the N58/K59 vs. Y58/E59 polymorphisms of CagL in isogenic mutants of the well-established GC strain 7.13 [19] during infection of gastric epithelial cells in vitro and of Mongolian gerbils in vivo.

## Materials and methods

### Genetic analyses of CagL in worldwide *H. pylori* strains

Previously, we created a dataset containing 2,267 *H. pylori* genomes of worldwide origin, for which information on the patient’s disease status is available [53]. The gene sequences of *atpA*, *efp*, *mutY*, *ppa*, *trpC*, *ureI*, and *yphC*, that are commonly used for multi-locus sequence analysis (MLSA), were extracted using blastn. The isolates and sequences of published reference strains [4, 5, 8] were assigned to the biogeographic *H. pylori* populations hpAfrica1, hpNEAfrica, hpNEAfrica, hpAsia2, hpEurope, hpEastAsia, and hpNorthAsia (hspAmerind) using Structure version 2.3.4 as described [4, 7]. Grouping of the isolates to known biogeographic *H. pylori* populations allowed analysis of the sequences based on genetic ancestry rather than by their geographic origin, which can be blurred by human population movements. The CagL protein and the *cagL* gene sequences were extracted using tblastn and blastn, respectively. The CagL protein was analyzed for the CagL hypervariable motif at amino acid positions 58 and 59 as well as amino acid positions 58 to 62. The *cagL* gene phylogeny was visualized as a Neighbor-joining tree constructed in MEGA version X [62]. The individual isolates were colour coded according to their population assignment. Pairwise Fisher’s exact tests were used to compare the allele frequencies of the CagL sequence motifs across the disease groups Mild (normal mucosa and non-atrophic gastritis), PUD (peptic ulcer disease) and GC. To this end, individual Fisher’s tests were run on every pair (https://www.socscistatistics.com/tests/fisher/default2.aspx). *P*-values of *P* < 0.05 were considered as statistically significant.

### *H. pylori* strains, CagL mutagenesis and genetic complementation

The gerbil-adapted *H. pylori* wild-type (wt) strain 7.13 (originally isolated as strain B128 from a patient with duodenal ulcer [19]), was grown from cryopreserved glycerol stocks. Cultivation was performed on gonococcal (GC) agar plates containing 10% donor horse serum (PAN Biotech GmbH, Aidenbach, Germany), 1% vitamin mix and selected antibiotics (colistin, vancomycin, trimethoprim and nystatin) at described concentrations [31, 43]. The bacteria were maintained for 48 h under microaerobic conditions by using an anaerobic jar equipped with a CampyGen sachet. Subsequently, the bacterial cells were resuspended in Brain Heart Infusion (BHI) medium and were subcultured for an additional 24 h on a fresh GC agar plate before further use. Mutagenesis and genetic complementation of the *cagL* gene was performed in 7.13 according to a detailed published protocol [63]. The corresponding cloning strategy is explained in Supplementary Figure S1 and primers in Supplementary Table S3. Four individual clones each were isolated for further characterization by PCR, sequencing and Western blotting.

### Molecular modeling

The structure of six different CagL polymorphisms was modelled in the background of strain 7.13 using AlphaFold 3 [64]. The comparison of the models was done with PDBeFold [65]. Structure analysis and visualization was performed with RasMol [66] and RasTop (https://www.geneinfinity.org/rastop/).

### Mongolian gerbil model of *H. pylori* infection

All animal studies were carried out in accordance with the *Guide for the Care and Use of Laboratory Animals* and with the Animal Care and Use Program at Vanderbilt University Medical Center (Nashville, Tennessee, USA). Following overnight fasting, Mongolian gerbils (*n* = 5 per group) were orogastrically infected with the four above-described *H. pylori* strains. Control animals (*n* = 5) were orogastrically challenged with Brucella broth (mock, treated with sterile broth medium alone). Twelve weeks post-challenge, the gerbils were euthanized and gastric tissue was harvested for quantitative *H. pylori* culture and histopathology, as previously described [67].

### *H. pylori* culture and density analysis

One longitudinal strip of gastric tissue from each animal was homogenized in sterile phosphate-buffered saline. Aliquots of serial dilutions were plated on selective trypticase soy agar supplemented with 5% sheep blood, vancomycin, nalidixic acid, amphotericin B, and bacitracin as previously reported [68]. Plates were incubated for five to seven days at 37 °C with 5% CO_2_. Colonies were identified as *H. pylori* based on characteristic morphology, Gram stain, urease, and oxidase activities. Colonization density is expressed as colony-forming units/gram of tissue (CFU/g) [68].

### Histopathology in infected Mongolian gerbils

The histopathology procedure used was described in detail [67]. Briefly, linear strips of gastric tissue, extending from the squamocolumnar junction to the proximal duodenum, were fixed in 10% neutral buffered formalin, embedded in paraffin, and tissue sections were stained with hematoxylin and eosin (H&E). A pathologist blinded to experimental conditions provided histopathological diagnoses (normal, gastritis, or adenocarcinoma) and assessed indices of gastric inflammation. Severity of acute and chronic inflammation was graded separately in both the antrum and the corpus in a scale 0–3 (0, no inflammation; 1, mild; 2, moderate; 3, marked inflammation) for a total combined score of 0–12 [69]. Acute inflammation is described as the infiltration of polymorphonuclear neutrophils (PMNs) and chronic inflammation as the infiltration of mononuclear leukocytes [69]. Histological diagnosis categories reflected the most advanced diagnosis. Gastritis was diagnosed in the presence of any (even minimal) level of inflammation and absence of adenocarcinoma. Dysplasia was observed in gerbils with adenocarcinoma, but not as the most advanced diagnosis in any of the animals. Adenocarcinoma was diagnosed according to published guidelines [70, 71], and was characterized by irregular glandular structures, with budding and branching, lined by epithelial cells with hyperchromatic and pseudostratified nuclei, and penetrating through the muscularis mucosae in the submucosa [70, 71].

### Infection of cultured AGS cells and elongation phenotype

For cell culture, AGS (ATCC CRL-1739) human gastric adenocarcinoma cells and polarized AGS cells stably transfected with wt human E-cadherin [72] were grown using Roswell Park Memorial Institute (RPMI) 1640 medium (Thermo Fisher Scientific, Waltham, Massachusetts, USA) containing 10% fetal bovine serum (FBS, Thermo Fisher Scientific), 1% penicillin-streptomycin (Sigma-Aldrich, Taufkirchen, Germany), and 0.2% Normocin (InvivoGen, Toulouse, France) at 37 °C and 5% CO₂. For infection experiments with *H. pylori*, the cultures were kept in 6-well plates until they reached approximately 70% confluency. Then, the cells were washed twice with PBS and transferred to plain, antibiotic-free RPMI-1640 medium to avert any antibiotic interference [73]. The cells were infected with *H. pylori* (prepared as detailed above) at multiplicity of infection *(*MOI) of 50 for the indicated time periods (1–24 h, see figure legends). Phenotypical changes and the number of elongated AGS cells upon infection were monitored via phase-contrast microscopy as previously described [38, 74].

### Scanning electron microscopy

Following infection, cells were fixed on ice for one hour using a mixture of 5% formaldehyde and 2% glutaraldehyde in HEPES buffer (0.1 M HEPES, 0.01 M CaCl_2_, 0.01 M MgCl_2_ and 0.09 M sucrose, pH 6.9). Two washing steps with TE buffer (10 mM TRIS, 2 mM EDTA) followed. The samples were then subjected to dehydration steps using acetone solutions at varying concentrations (10%, 30%, 50%, 70%, and 90%) for 30 min per step, followed by two 10 min incubations in 100% acetone at room temperature. Critical point drying was performed using Leica’s automated system (CPD 300) and the samples were then sputter-coated with gold/palladium. After mounting the samples on 12 mm aluminium stubs with adhesive tape, observations were conducted using a Zeiss Merlin field-emission scanning electron microscope at an acceleration voltage of 5 kV, and the images were acquired in a 75:25 ratio using the inlens-SE detector and the Everhart-Thornley SE detector.

### Quantification of bacterial cell binding

To examine bacterial adhesion, AGS cells that had been seeded and had reached 75% confluency in 6-well plates were rinsed with prewarmed, antibiotic-free RPMI-1640 medium and were infected with the specified *H. pylori* strains (MOI = 50) for 6 h. After three washing steps with medium, the AGS host cells were selectively lysed by adding 1 mL of 0.2% saponin solution (50 mM Tris, 1 mM NaF, 0.4 mM Na₃VO₄, 0.2% saponin, 1× cOmplete™ protease cocktail) at 37 °C for 20 min. Viable adherent bacteria were quantified by plating serial dilutions of the resulting lysates onto GC agar plates. After 3 days of incubation at 37 °C under microaerophilic conditions, *H. pylori* colonies were counted to determine colony-forming units (CFUs).

### Wound healing assay

To assess cell migration by wound healing, we followed published protocols [75]. For this purpose, AGS cells were grown to confluency in 6-well plates. A mechanical wound was generated across the monolayer using a pipette tip, followed by two rinsing steps with fresh RPMI-1640 medium to eliminate detached cells. The wounded monolayers were then challenged with the various *H. pylori* strains (MOI = 50) for 48 h. Microscopic evaluation of wound healing was performed by phase-contrast microscopy using the Leica DMI4000B system. Cell migration was quantified by analysing the reduction of the wound area over time.

### NF-κB quantification and IL-8 ELISA

NF-κB transcription factor activity was assessed via a secreted embryonic alkaline phosphatase (SEAP) reporter assay using AGS cells [52]. To this end, cells were seeded in 6-well plates until they reached 70–90% confluency, followed by transfection with 4 µg pNF-κB-SEAP plasmid (Addgene) for 48 h utilizing TurboFect™ (Thermo Fisher Scientific). The cells were then infected with *H. pylori* (MOI = 50) for 24 h and NF-κB-dependent SEAP production was quantified by mixing 20 µL of infected culture supernatant or mock-treated control medium with 180 µL of QUANTI-Blue reagent (InvivoGen) in 96-well plates, followed by incubation at 37 °C for 30 min. Absorbance was measured at 620 nm using an Infinite F200 Pro microplate reader (Tecan, Grödig, Austria). Secreted IL-8 concentrations were determined from the same supernatants using a commercially available ELISA kit (Invitrogen, #88-8086) as previously described [76].

### Beta-catenin activation

The methodology was basically performed as described previously [19]. Briefly, to evaluate β-catenin-dependent TCF transcriptional activity, the reporter plasmid constructs TOPflash and FOPflash (Merck-Millipore, Darmstadt, Germany) were used. TOPflash serves as a T-cell factor (TCF)-responsive reporter, containing six wt TCF binding sites (two sets of three copies each) upstream of a minimal thymidine kinase promoter that drives a luciferase reporter gene. As a negative control, FOPflash contains mutated TCF binding sites upstream of the same promoter-luciferase cassette and was used to account for background transcriptional activity.

### DNA damage analysis

To assess DNA fragmentation in the host cells, total DNA was extracted from *H. pylori*-infected or non-infected AGS cells. The resulting DNA samples were separated via pulsed-field gel electrophoresis (PFGE) as previously described [52, 77]. After staining the gels with ethidium bromide, the gel images were captured under UV light using a ChemiDoc documentation system, and the respective band intensities were densitometrically analysed with ImageLab software (Bio-Rad Laboratories, Hercules, California, USA).

### Western blotting

For protein analysis, bacterial and cell lysates prepared in SDS sample buffer (5 min boiling) were resolved on 6–12% SDS-PAGE gels. Gels were either stained directly with Coomassie Brilliant Blue (Merck, Darmstadt, Germany) or the separated proteins were transferred to polyvinylidene difluoride (PVDF) membranes (Carl Roth, Karlsruhe, Germany) for Western blotting analysis. PVDF membranes were blocked with 5% skim milk or 3% BSA in TBST buffer [73], followed by incubation (2 h, RT) with the primary antibodies listed below to detect the respective target proteins: rabbit anti-CagA (Austral Biologicals, #5003-9); rabbit anti-phospho-CagA (Y-972) [38]; rabbit anti-CagL [38]; rabbit anti-phospho-ERK1/2 (T202/Y204) (Cell Signaling, #CS9101S); rabbit anti-phospho-JNK/SAPK (T183/Y185) (Cell Signaling, #CS9251); anti-phospho-p38 (T180/Y182) (BD, #612280) and mouse anti-GAPDH (Santa Cruz, #sc-47724) for detection of glycerinaldehyd-3-phosphat-dehydrogenase, rabbit anti-CagY [6] and rabbit anti-urease B (UreB) [78] as controls. Bound antibodies were visualized by using horseradish peroxidase (HRP)-conjugated goat anti-mouse (#31446) or goat anti-rabbit (#31462) secondary antibodies (Thermo Fisher Scientific) and enhanced chemiluminescence (ECL) detection solution (comprised of 100 mM Tris-HCl [pH 8.6], 2.5 mM luminol, and 0.4 mM p-coumaric acid, mixed with hydrogen peroxide).

### Statistics

All statistical valuations were conducted via GraphPad Prism (software version 8.0) using data from at least three independent experimental runs (biological replicates) presented as mean ± standard deviations (SD). Prior to statistical testing, the intensity of Western blot bands was quantified via Image Lab software (version 6.1.0; Bio-Rad) and relative values calculated based on the maximum intensity (100%). For standard comparative analyses, statistical significance was calculated using one-way ANOVA followed by Tukey’s multiple comparison test. A value of *p* ≤ 0.05 was defined as the threshold for statistical significance.

## Results

### Analysis of the CagL and gastric disease status from 2,267 worldwide *H. pylori* strains

**Fig. 1:**
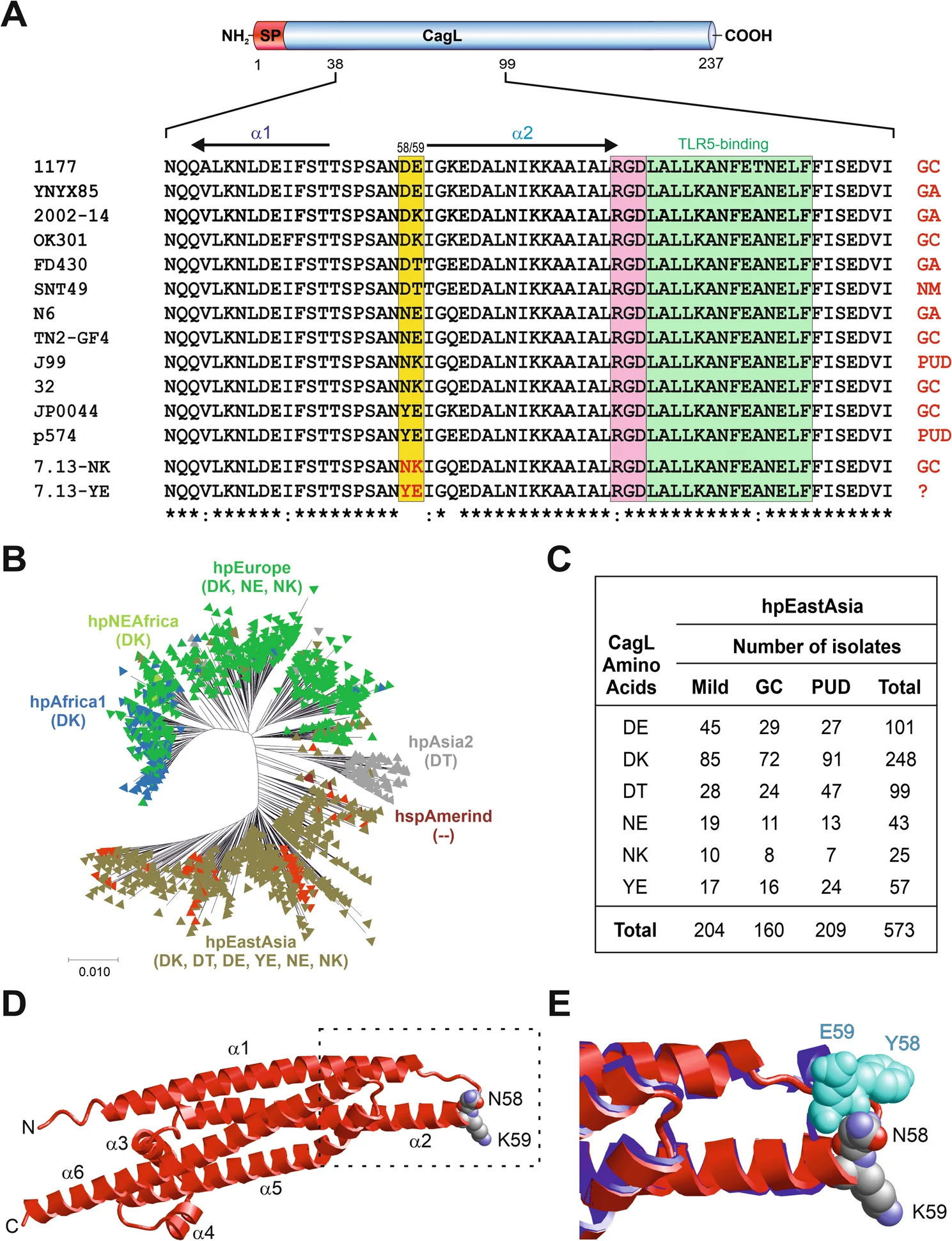
A hypervariable motif in the T4SS-pilus protein CagL of *H. pylori* and its association with gastric disease. **A** Graph depicting the CagL protein (blue) with a signal peptide (SP, red) at the N-terminus. The position of the hypervariable motif at position 58/59 (yellow box), an RGD motif (violet) and a TLR5 binding site (green) in CagL are marked. Sequence alignment of the six most frequently detected hypervariable sequence motifs, two representatives each are shown, plus the 7.13 wt strain (7.13-NK) and the isogenic 7.13-YE mutant (bottom). The specific stomach condition is listed on the right. Abbreviations used: GC (gastric cancer), PUD (peptic ulcer disease), GA (gastritis), NM (normal mucosa). **B** Neighbour joining tree showing the phylogeny of the *cagL* gene and the phylogeographic distribution of the CagL motifs in isolates of the color-coded biogeographic populations of *H. pylori*. The YE motif (red label) was only identified among isolates of the population hpEastAsia. **C** Frequency and association with gastric disease of the six most common CagL motifs in hpEastAsia strains. Mild: normal mucosa and non-atrophic gastritis. **D** Model of the CagL structure from strain 7.13. Residues N58/K59 at the site of the polymorphism are shown in space-filled presentation (coloured by atom type). The α-helices and the termini are labelled. The dotted part of the structure is analysed in more detail in panel E. **E** Enlargement of the N58/K59 motif region overlaid with a CagL model (blue) harbouring the Y58/E59 motif. The Y58/E59 motif highlighted in cyan exhibits a significantly different conformation compared to the N58/K59 of strain 7.13

Our recently compiled dataset of 2,267 *H. pylori* genomes from around the world, for which the disease status of the patient is known [53], was analyzed for the *H. pylori cagL* gene. This analysis revealed the presence of 1,743 *cag*PAI T4SS-positive *H. pylori* strains and 524 *cag*PAI T4SS-negative isolates. As expected, the genomes of *cag*PAI-negative strains did not contain the *cagL* gene. The 1,743 *cagL* genes from *cag*PAI-positive isolates were extracted from the genomes. Of those, eight *cagL* genes were truncated, and thus incomplete, and were excluded from the analysis. The remaining 1,735 full-length *cagL* sequences were acquired from patients with mild gastric disease (normal mucosa and non-atrophic gastritis, Mild, *n* = 827), atrophic gastritis (AG, *n* = 135), metaplasia (Meta, *n* = 161), gastric cancer (GC, *n* = 282), MALT (*n* = 18), and peptic ulcer disease (PUD, *n* = 312). The CagL hypervariable motif at position 58/59, which is located N-terminal of the integrin-binding RGD motif [38] in a loop between alpha helices α1 and α2 (Fig. 1A), contained 16 distinct amino acid sequence motifs (Supplementary Table S1). The most frequent amino acid motifs were D58/K59 (DK) in 738 isolates, NE (*n* = 321), NK (*n* = 216), DT (*n* = 209), DE (*n* = 111), YE (*n* = 75), and -K (*n* = 36). Other motifs were very rare and were detected in only few *H. pylori* isolates. We assigned the individual isolates to the known biogeographic *H. pylori* populations hpEastAsia, hpAsia2, hpEurope, hpAfrica1, hpNEAfrica, and hspAmerind. This enabled us to analyse the sequences on the basis of their genetic ancestry rather than their geographic origin, which can sometimes result in a blurred picture due to human migration patterns. For example, isolates assigned to the population hpEurope were not only isolated from patients in Europe and the Middle East, but also from other geographic regions that were colonised by people carrying hpEurope bacteria, including Central and South Asia, North America, South America, Australia, and South Africa. Likewise, hpAfrica1 isolates were also isolated in the Americas. Isolates assigned to the African populations (hpAfrica1, hpNEAfrica) contained DK, the five exceptions were isolated elsewhere (USA, Mauritius) and are attributed to recent admixture and random drift by point mutation (Supplementary Table S1). Analysis of 70 additional hpNEAfrica genomes of isolates from Ethiopia, Nigeria, Somalia and Sudan without patient disease data confirmed the sole presence of the DK allele. Isolates of the hpAsia2 population contained almost only DT, and hpEurope isolates frequently possessed DK, NE or NK. *H. pylori* from indigenous Americans (hspAmerind) contained a gap of the two amino acids (--) at this position. The greatest diversity was observed among isolates of the population hpEastAsia, with the most common motifs being DK, DE, DT, NE, NK, and YE (Fig. 1B**/**C, Supplementary Table S1). Similar observations regarding putative ancestral alleles in Africa and the greatest sequence diversity in East Asia were published by Gorrell and colleagues on a smaller dataset [59]. Thus, the motif YE that was previously reported to be associated with increased risk of GC [55], was only present in a small subpopulation of hpEastAsia (Fig. 1B/C, Supplementary Table S1). However, none of the CagL motifs showed a significant correlation with GC compared to mild disease (*p* values ranging from *p* = 0.3944 to *p* = 1). Yet, compared to DE, DT and YE were significantly associated with PUD (*p* = 0.002953 and *p* = 0.048665, respectively). Similarly, there was no disease association of the CagL motifs among the hpEurope isolates.

Expanding the analysis to the five amino acids at positions 58 to 62 increased the dataset to 43 genotypes of the CagL hypervariable motifs (Supplementary Table S2). However, the sequences showed no significant correlation with gastric disease. Similar to the previous publication by Gorrell and co-workers [59], the codon usage at the CagL hypervariable amino acid positions 58–62 was highly conserved, with only 30 of the 8,675 codons representing synonymous mutations across the 1,735 isolates. In this expanded dataset, novel variants were discovered at position 59 that were not previously described, namely four glycine (codon GGA), three arginine (AGA), two glutamine (CAA) and one isoleucine (ATA). Among the 30 synonymous changes, 27 GGT → GGC changes occurred at position 61 that does not appear to be disease associated. Isolates carrying this mutation were identified among hpEurope isolates from Sweden (17 of 136 isolates), France (1 of 34), Greece (1 of 25), Belarus (1 of 2), Australia (1 of 18), 1 out of 8 hpAfrica1 isolates from Colombia, as well as hpEastAsia isolates from China (1 of 326), and Japan (1 of 166). Overall, all other genetic changes were non-synonymous, suggesting selection.

Modeling of the CagL structure from strain 7.13 (Fig. 1D) exhibited the typical helix structure as already known from the crystal structures of CagL from other strains. Modelling of the structures of different CagL polymorphisms in the isogenic background of strain 7.13 exposed some distinct structural variations at the site of polymorphism, which may affect the functional properties. These differences were most pronounced between the structures containing the NK and YE motifs (Supplementary Figure S2). A structural overlay shows that residues 58–59 adopt distinct conformations in the NK and YE motifs (Fig. 1E).

### *H. pylori* colonization of Mongolian gerbil mucosa and development of gastric disease

**Fig. 2:**
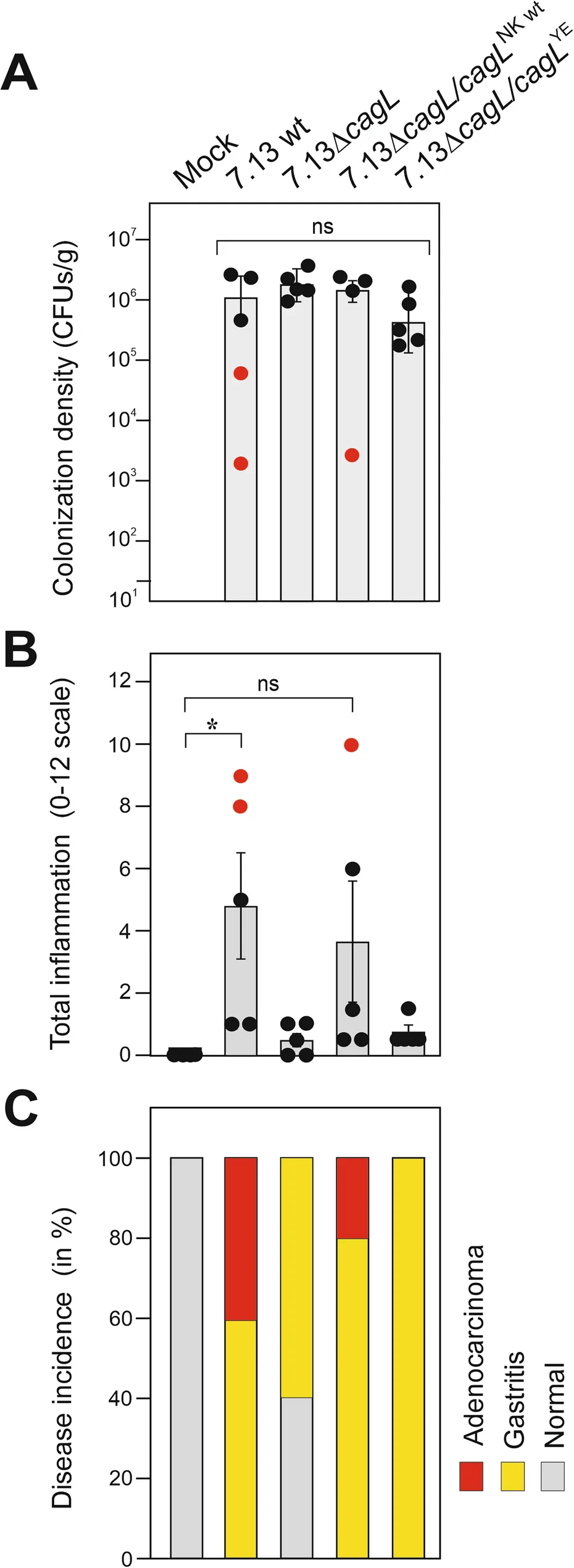
Colonization density, inflammation score and histopathology of Mongolian gerbils after gastric infection with *H. pylori.* Five gerbils each were inoculated with Brucella broth (Mock) or infected with the indicated *H. pylori* strains for 12 weeks. **A** Gastric tissue was homogenized and plated on selective trypticase soy agar plates with 5% sheep blood for quantification of viable *H. pylori*. Colonization density is expressed as colony-forming units per gram of gastric tissue (CFU/g). Each point represents data from an individual animal; red points correspond to gerbils with adenocarcinoma. Mean values are shown in scatter dot plots with standard error of the mean (SEM). A one-way ANOVA with a post hoc Tukey’s test was used to determine statistical significance. (*, *p* < 0.05); all other comparisons were not significant). **B** Determination of total gastric inflammation scores. Each data point represents an individual animal; red points correspond to gerbils with adenocarcinoma. Mean values are shown in scatter dot plots with SEM. A one-way ANOVA with a post hoc Tukey’s test was used to determine statistical significance. (*, *P* < 0.05; ns, not significant). **C** Histopathology was classified as normal, gastritis, or adenocarcinoma, and represents the most advanced lesion observed within the gastric tissue section

**Fig. 3:**
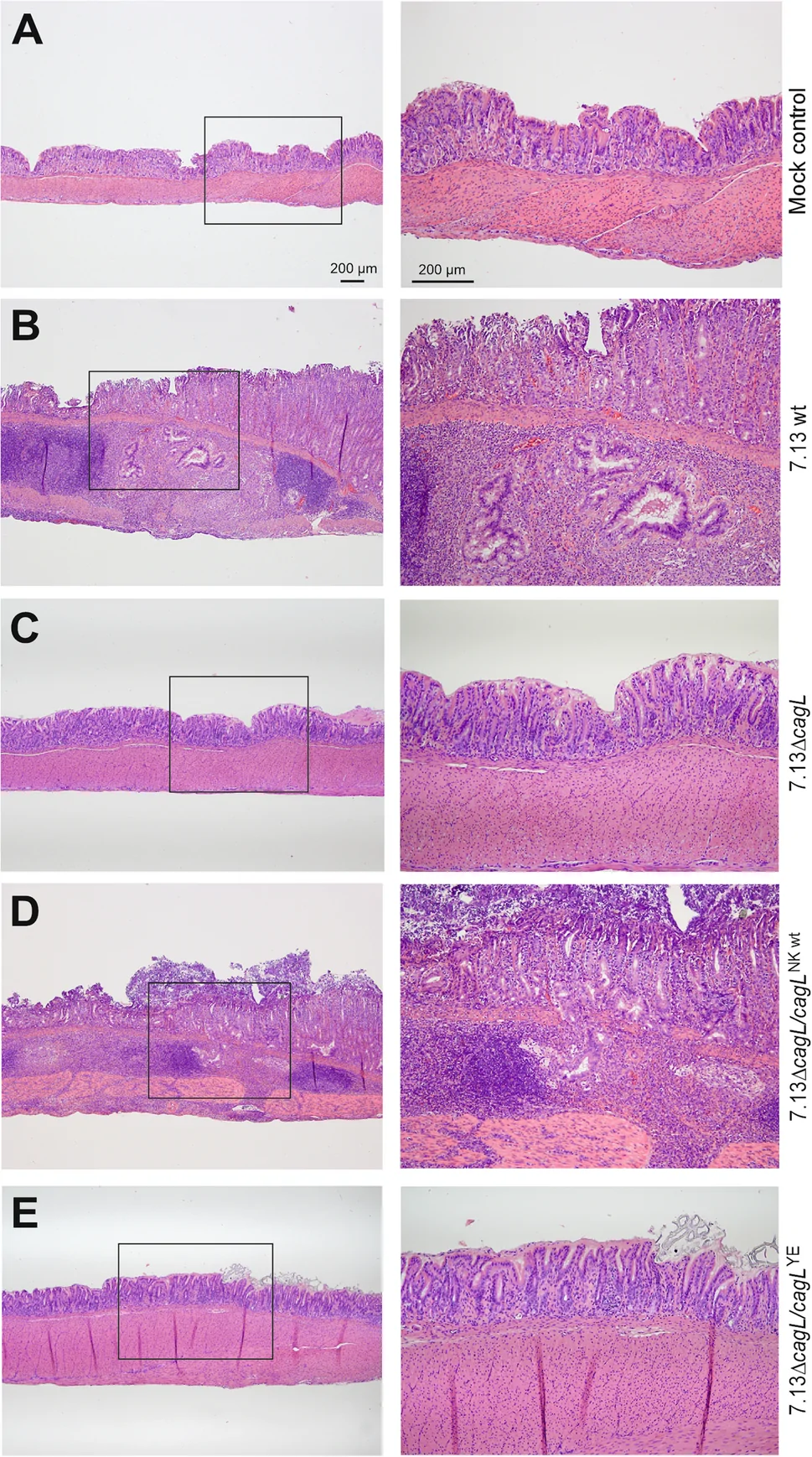
Histopathological examination of the gastric mucosa in Mongolian gerbils after 12-week infection with *H. pylori.* Hematoxylin & eosin (H&E) stained linear strips of gastric tissue, extending from the squamocolumnar junction to the proximal duodenum. Representative photomicrographs of (**A**) normal stomach tissue from an uninfected gerbil, marked inflammation and adenocarcinoma in gerbils infected with 7.13 wt (**B**) or the CagL complemented strain (**D**). The other panels show attenuated host responses in gerbils infected with the Δ*cagL* deletion mutant (**C**) or the 7.13 strain carrying the YE allele of CagL (**E**). Boxes in the left picture mark the positions of enlarged sections shown to the right. Pictures have been taken at 40x (left) and 100x (right) magnification. All scale bars represent 200 micrometers

Next, we aimed to study the importance of the NK and YE polymorphisms of CagL in more detail. We utilized the previously reported GC strain 7.13, which is capable to induce GC in Mongolian gerbils within 12 weeks of infection [19]. For this purpose, we generated isogenic *H. pylori* 7.13 variants, named 7.13Δ*cagL* deletion mutant, 7.13Δ*cagL* complemented with *cagL* wt with the NK allele (7.13Δ*cagL/cagL*^NK wt^), and 7.13Δ*cagL* complemented with the isogenic YE allele (7.13Δ*cagL/cagL*^YE^). We have checked four individual clones each of our complemented strains. No differences were seen among these strains regarding the expression and band sizes of T4SS proteins CagL, CagA and CagY with urease B as loading control, except for the lack of the CagL band in the Δ*cagL* deletion mutant as expected (Supplementary Figure S3), and no phenotypical differences among the four individual NK or YE clones upon infection of AGS gastric epithelial cells in vitro (data not shown). These *H. pylori* strains, along with one representative clone of the CagL NK or YE alleles, were perorally infected for 12 weeks with 10^9^ viable bacteria into five Mongolian gerbils each. All *H. pylori*-challenged animals were successfully colonized by all strains (Fig. 2A). Although *H. pylori* re-isolation was not detected in one of the gerbils inoculated with strain 7.13Δ*cagL/cagL*^NK wt^, this animal developed antral gastritis, and colonization was confirmed by histology. Notably, gerbils that developed adenocarcinoma showed somewhat lower colonization densities than gerbils that only developed gastritis within their respective groups, but these differences were statistically not significant (red circles in Fig. 2A). As expected, *H. pylori* were not isolated from uninfected gerbils. Total gastric inflammation was assessed in H&E-stained sections using our established scoring system ranging from 0 to 12 [69]. Corresponding H&E sections from each Mongolian gerbil cohort are shown in Fig. 3A-E. Compared with uninfected gerbils (mock controls), animals infected with 7.13 wt exhibited significantly higher levels of gastric inflammation (*p =* 0.02), followed by the complemented ∆*cagL/cagL*^NK^ mutant (Fig. 2B). No significant differences in total gastric inflammation were observed between mock-treated gerbils and animals infected with the ∆*cagL* mutant. In general, minimal levels of inflammation were observed in animals infected with ∆*cagL/cagL*^YE^ mutant strain. Individual Mongolian gerbils that developed adenocarcinoma displayed the highest levels of inflammation in their respective groups and were marked with red circles (Fig. 2B). Regarding histological diagnosis (Fig. 2C), adenocarcinomas were observed in 40% (2/5) of animals infected with 7.13 wt and 20% (1/5) of those infected with the complemented ∆*cagL/cagL*^NK^ strain. Normal mucosa was observed in 40% (2/5) of the animals infected with the ∆*cagL* deletion mutant and 100% (5/5) in mock-treated animals. Together, these data suggest that *H. pylori* 7.13 expressing wt CagL^NK^ induced severe gastric pathology and even adenocarcinoma, whereas the CagL^YE^ mutant revealed a largely attenuated phenotype with no adenocarcinoma.

### Elongation phenotype and CagA phosphorylation during infection of AGS cells

**Fig. 4:**
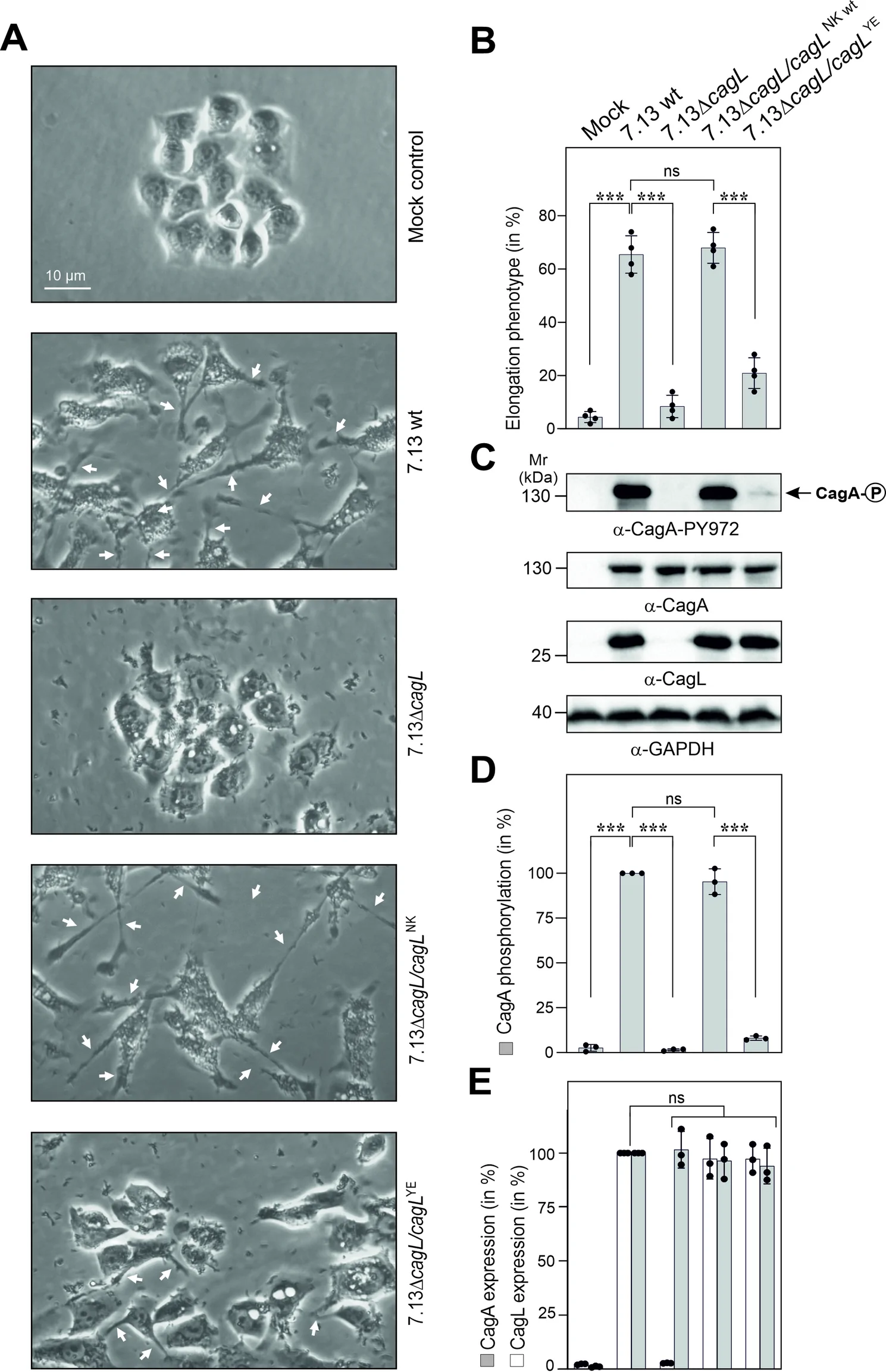
Phase contrast microscopy, CagA phosphorylation and CagL expression during *H. pylori* infection of AGS gastric cells. **A** Phase contrast microscopy images of cells that were infected for 6 h with the indicated *H. pylori* strains. Examples of elongated AGS cells are marked with white arrows. **B** Quantification of elongated cells presented as mean values ± standard deviation (SD) from at least three independent experiments. **C** Western blots of cell lysates after infection. The phospho-specific α-CagA-PY-972 was used to detect phosphorylated CagA. The α-CagA and α-CagL blots detect total CagA and CagL proteins in the samples, respectively. The GAPDH blot served as loading control for host cell proteins. **D** Quantification of phospho-CagA band intensities by densitometry. **E** Quantification of total CagA and CagL band intensities by densitometry. Each point represents data from an individual experiment. All graphs display mean values ± SD derived from a minimum of three or four independent biological replicates as indicated. The p-value *p* ≤ 0.001 (***) was defined as statistically significant. ns – not significant

In the next set of experiments, we aimed to study phenotypical changes during infection of cultured AGS cells. In particular, *H. pylori* was reported to induce the cancer-associated cell scattering and elongation phenotype in a T4SS- and CagA-dependent fashion [74]. To this end, we used AGS cells at about 70% confluency that were infected with the above strains for 6 h, and phenotypical changes were monitored by phase contrast microscopy (Fig. 4A**)**. The results show that *H. pylori* 7.13 wt and complemented ∆*cagL/cagL*^NK^ mutant displayed the highest number of scattered and elongated cells (Fig. 4A**/**B). In contrast, Δ*cagL* deletion and ∆*cagL/cagL*^YE^ point mutants revealed a strong downregulation of AGS cell elongation. Quantification data showed that the ∆*cagL/cagL*^YE^ mutant exhibited a significant reduction, suggesting an attenuated phenotype as seen in the infected Mongolian gerbils (Fig. 4A**/**B).

Next, AGS cells from the same experiment were harvested after 6 h infection and cell lysates were examined for the presence of phosphorylated CagA. Western blotting using an antibody against phosphorylated CagA (α-CagA-PY972) showed strong bands for CagA phosphorylation in *H. pylori* 7.13 wt and complemented ∆*cagL/cagL*^NK^ mutant, respectively (Fig. 4C, top). The Δ*cagL* deletion mutant showed no band of phospho-CagA, while the ∆*cagL/cagL*^YE^ point mutant revealed a faint band (Fig. 4C). In agreement with the induction of cell elongation, quantification of phospho-CagA band intensities revealed no statistic difference between 7.13 wt and *cagL*^NK^ mutant, but a 12-fold reduced phospho-CagA in the *cagL*^YE^ mutant, confirming an attenuated phenotype (Fig. 4D). Control blots for total CagA and CagL expression showed equal levels of CagA and CagL proteins in each strain, with the exception of the Δ*cagL* deletion mutant that exhibited no CagL band as expected (Fig. 4C**/**E). The GAPDH blot served as loading control for host cell proteins (Fig. 4C).

### CagL NK- and YE-independent cell binding and T4SS-pilus formation

**Fig. 5:**
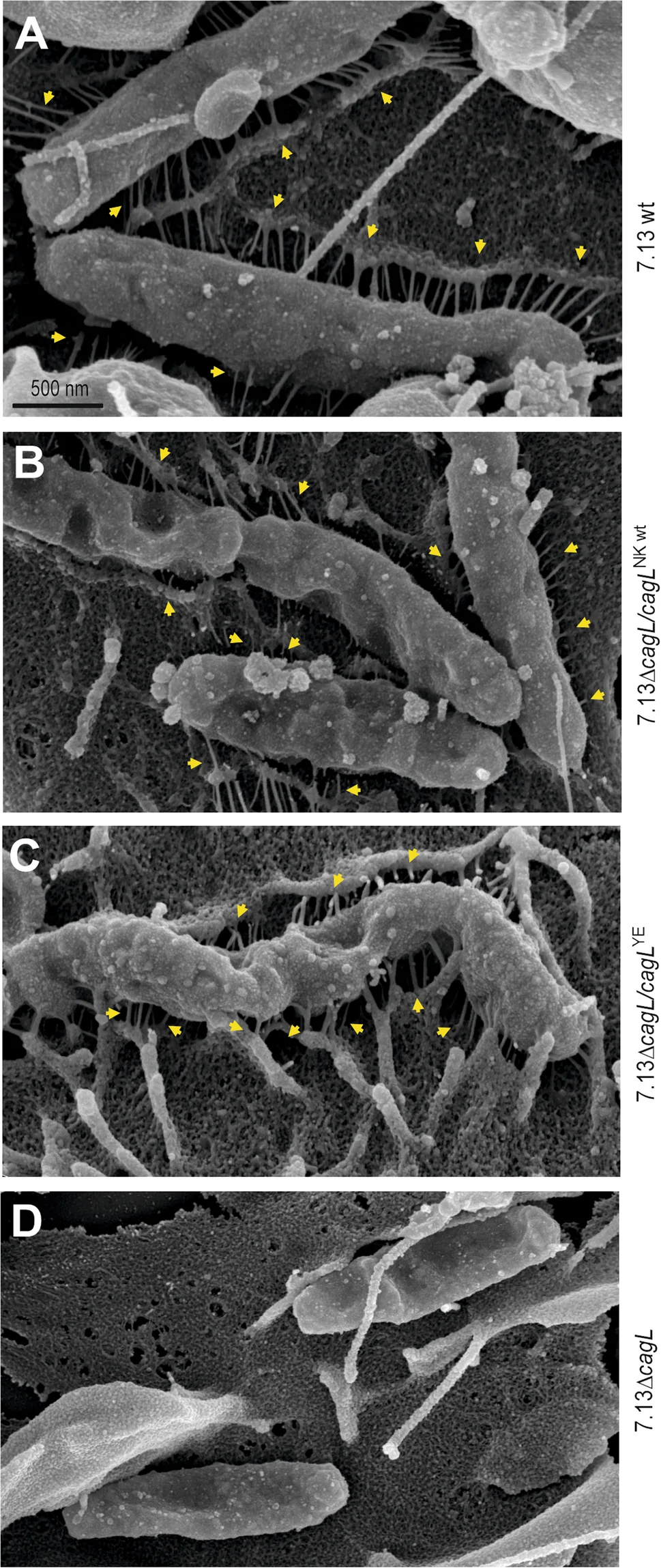
Scanning electron microscopy of T4SS-pilus formation by *H. pylori* CagL variants upon infection of AGS cells. AGS wt cells were infected for 6 h with the following *H. pylori* strains using an MOI of 10: (**A**) 7.13 wt, (**B**) 7.13Δ*cagL*/*cagL*^NK wt^, (**C**) 7.13Δ*cagL*/*cagL*^YE^, and (**D**) 7.13Δ*cagL*, followed by the preparation for scanning electron microscopy. The results show prominent formation of T4SS-pili for all CagL-expressing bacteria (examples marked with yellow arrows), but not the Δ*cagL* deletion mutant

The above results concerning the differential CagA phosphorylation by the *H. pylori cagL*^NK^ and *cagL*^YE^ variants were surprising. We therefore included two control experiments to exclude defects in cell binding and T4SS-pilus formation. First, we investigated *H. pylori* attachment to AGS cells by a bacterial cell binding assay. The results show similar cell binding rates, confirming that differences in CagA injection and phosphorylation were not due to varying host cell binding capabilities of the strains (Supplementary Figure S4). Second, we subjected infected AGS cell samples to scanning electron microscopy. In agreement with the above cell binding assay, we also observed homogeneous *H. pylori* binding to the AGS cell surface (Fig. 5 and data not shown). In addition, we found that *H. pylori* wt, *cagL*^NK^ and *cagL*^YE^ variants exhibited pronounced T4SS-pilus formation (Fig. 5A-C, examples are marked with yellow arrows), while the Δ*cagL* deletion mutant did not express any T4SS-pili (Fig. 5D) as expected [38]. These data implicate that all CagL-expressing *H. pylori* strains, including the *cagL*^YE^ variant, effectively bind to AGS cells and form T4SS-pili.

### CagL-dependent wound healing during *H. pylori* infection

**Fig. 6:**
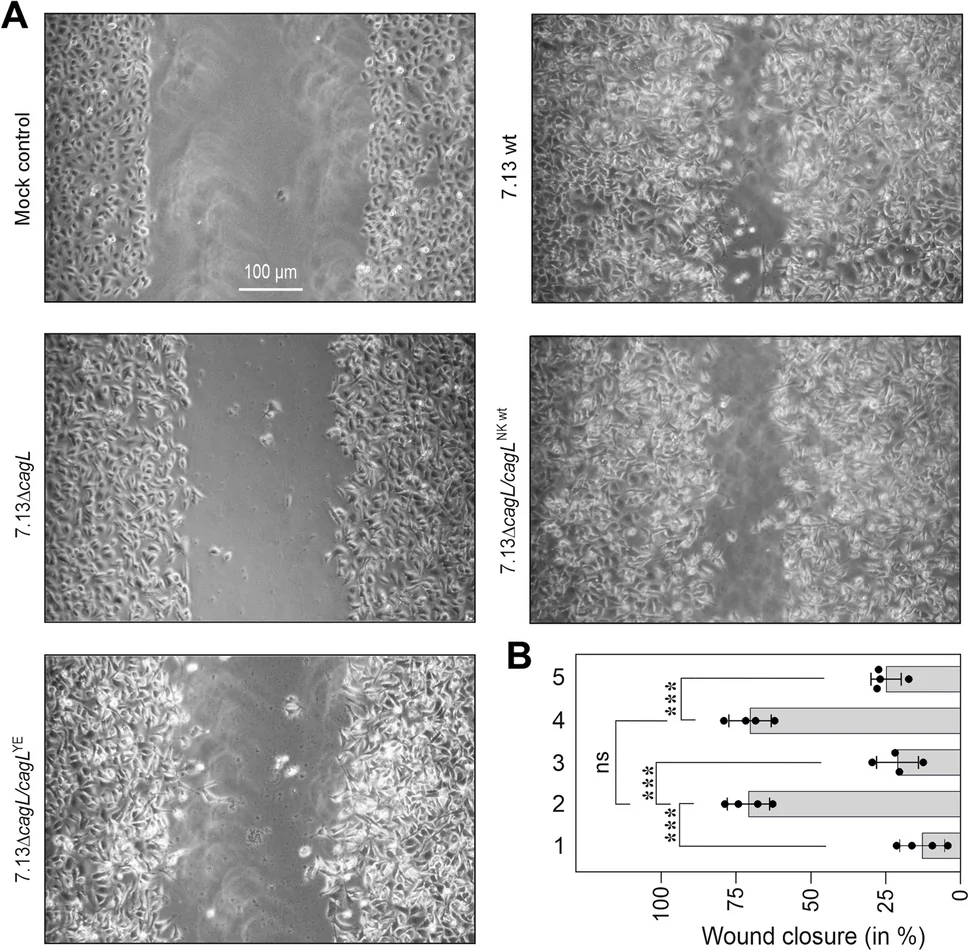
Induction of host cell motility during *H. pylori* of polarized AGS cells by wound healing assay. **A** Monolayers of polarized AGS cells stably transfected with wt E-cadherin were grown to 100% confluency. Then, the cells were wounded using a pipette tip, followed by infection for 48 h with the indicated *H. pylori* strains or treatment with medium as control (Mock). **B** After infection, the areas following wound closure were measured. 1 - Mock; 2–7.13 wt; 3–7.13Δ*cagL*; 4 -7.13Δ*cagL*/*cagL*^NK wt^; 5–7.13Δ*cagL*/*cagL*^YE^. Each point represents data from an individual experiment. All graphs display mean values ± SD derived from four independent biological replicates. *p*-values of ≤ 0.001 (***) were considered as statistically highly significant. ns – not significant

Collective cell migration is essential for tissue development and repair, as well as cancer metastasis [79]. The so-called wound healing assay is a standard two-dimensional method to measure cell migration and wound closure. The key steps involve creating a cell-free gap in a confluent cell monolayer, tracking cell movement over time, and analysing images to calculate the rates of gap closure. In the following experiments, we investigated the induction of cell migration (wound healing) induced by the various *H. pylori* strains. To this end, polarized AGS cells were grown to 100% confluency followed by setting a scratch-wound. These cells were then infected with *H. pylori*, and the wounded area was monitored by light microscopy over 48 h (Fig. 6A**/**B). Compared to the mock control, infection with *H. pylori* 7.13 wt and Δ*cagL/cagL*^NK^ mutant showed highly induced cell motility and effective closing of the wound. In contrast, the Δ*cagL* deletion and Δ*cagL/cagL*^YE^ mutants significantly reduced wound closure compared to the wt (Fig. 6A**/**B). Collectively, these data demonstrate that 7.13 wt and Δ*cagL/cagL*^NK^ mutant progressively stimulate AGS cell motility and wound closure, while Δ*cagL* deletion and Δ*cagL/cagL*^YE^ point mutation negatively regulated these responses upon infection.

### Activation of MAP kinases is controlled by CagL polymorphisms

**Fig. 7:**
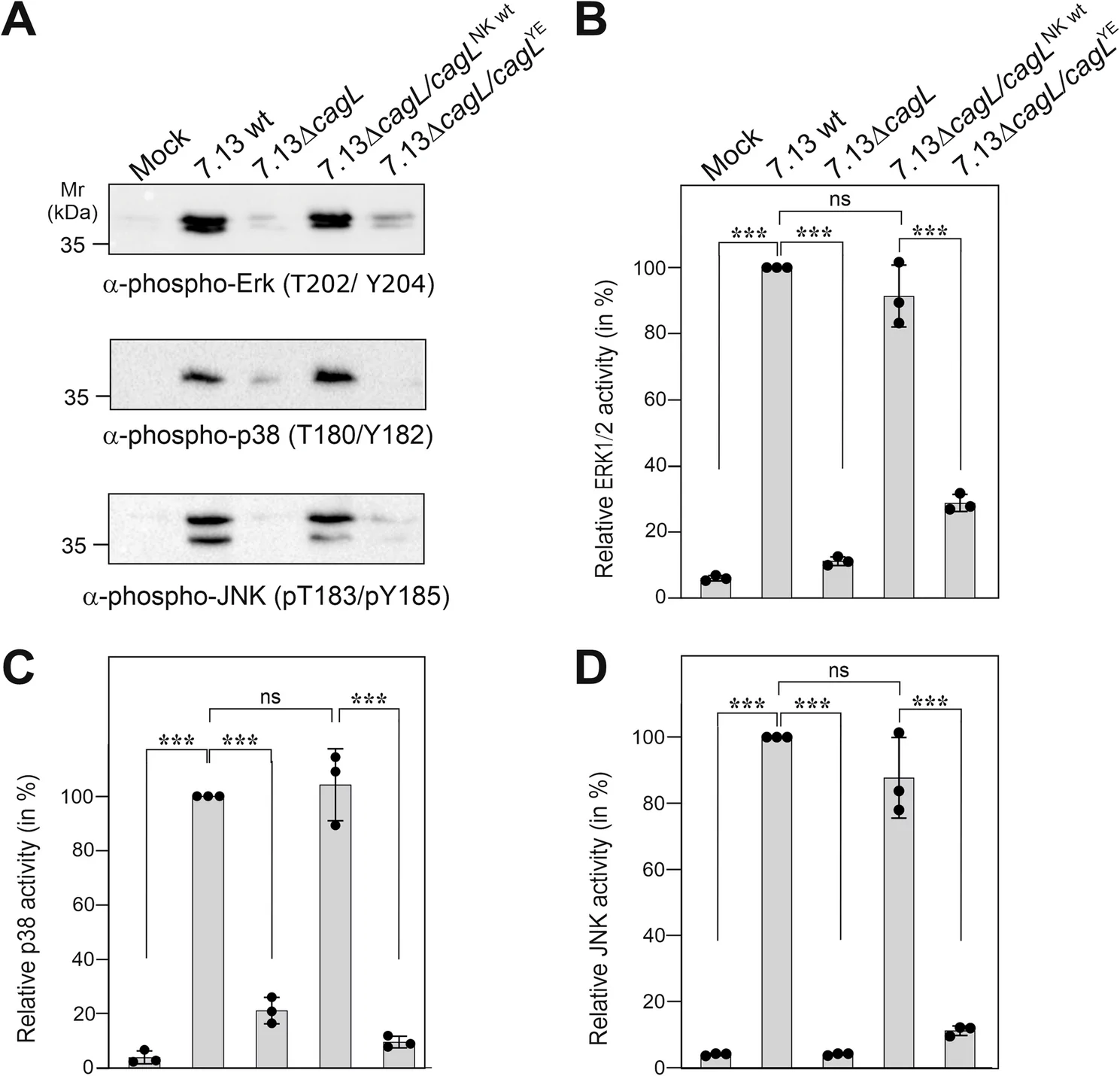
ERK1/2, p38 and JNK MAP kinase activation during *H. pylori* infection. MAP kinase activation is an early response by infected epithelial cells. **A** Starved AGS cells were infected with the indicated *H. pylori* strains for 60 min. After infection, cell lysates were prepared and subjected to Western blotting using the indicated phospho-specific MAP kinase antibodies. Quantification of phospho-band intensities by densitometry for phosphorylated ERK1/2 (**B**), phosphorylated p38 (**C**) and phosphorylated JNK (**D**), respectively. Each point represents data from an individual experiment. All graphs display mean values ± SD derived from three independent biological replicates. *p*-values of ≤ 0.001 (***) were considered as statistically highly significant. ns – not significant

Mitogen-activated protein (MAP) kinases are part of highly conserved signalling cascades that control the cell cycle, proliferation and differentiation [80]. Major mammalian MAP kinases comprise the ERK, JNK, and p38 MAP kinases, which translate external signals into intracellular responses via a multi-step signalling cascade. Finally, the activated MAP kinases migrate to the cell nucleus, where they phosphorylate and activate transcription factors [25, 80]. Thus, we next investigated whether the above *H. pylori* strains may differentially regulate the activation of MAP kinases (Fig. 7). To this end, we prepared lysates from *H. pylori-*infected AGS cells and subjected them to Western blotting using phospho-specific antibodies for ERK1/2, JNK and p38 (Fig. 7A). Compared to the mock control, infection with *H. pylori* 7.13 wt and Δ*cagL/cagL*^NK^ mutant showed highly induced ERK, JNK, and p38 phosphorylation (Fig. 7A-D). Conversely, the Δ*cagL* and Δ*cagL/cagL*^YE^ mutants showed significantly reduced MAP kinase phosphorylation (Fig. 7A-D). Together, these results show that 7.13 wt and Δ*cagL/cagL*^NK^ mutant progressively stimulate MAP kinase activation, while the Δ*cagL* deletion and Δ*cagL/cagL*^YE^ point mutants did not.

### Pro-inflammatory NF-κB activation and IL-8 release

**Fig. 8:**
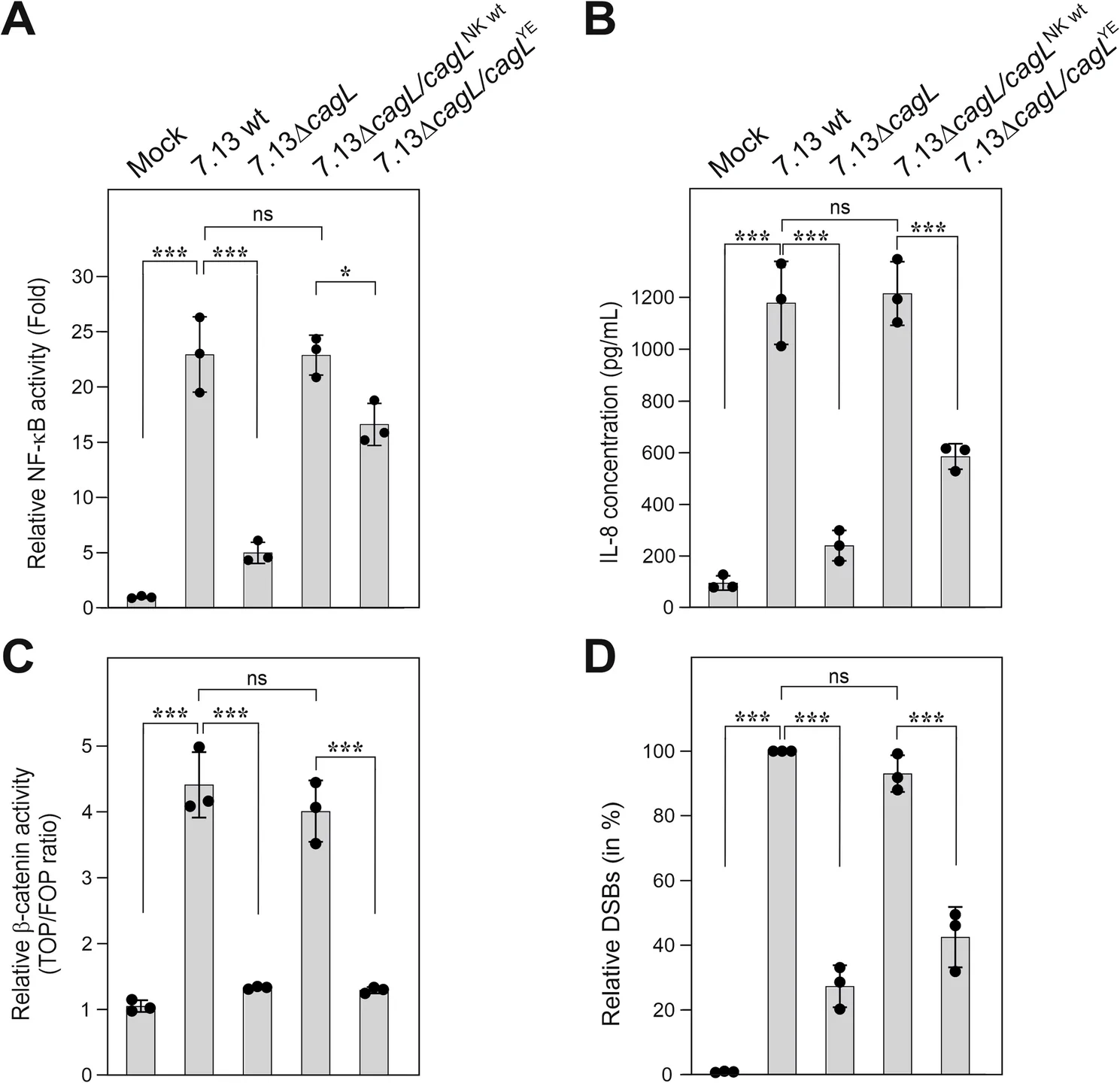
Activation of transcription factors NF-κB and β-catenin, IL-8 secretion and induction of chromosomal DNA damage in AGS cells upon *H. pylori* infection. Polarized AGS cells stably expressing E-cadherin were infected for 8 h with the indicated *H. pylori* strains and subjected to the following assays: (**A**) NF-κB activation by Quanti-Blue assay, (**B**) secretion of IL-8 measured by standard ELISA, (**C**) β-catenin activation by TOP/FOP luciferase assays, (**D**) PFGE of genomic host cell DNA. Quantification shows fragmented DNA in the gels that was normalized to the intact DNA retained in the upper wells, and to the uninfected mock control (set as 100%). Each point represents data from an individual experiment. Data are represented as mean ± SD from three independent biological replicates. Statistical significance was defined by *p* < 0.001 (***). ns – not significant

The transcription factor NF-κB (Nuclear Factor kappa-light-chain-enhancer of activated B cells) is a master regulator of inflammation, and other immune responses as well as cell survival [25]. The activation of NF-κB and chronic inflammation is a hallmark upon infection with *H. pylori* and mediated by the delivery of ADP-heptose and CagA [27, 28, 81]. To study the impact of the CagL polymorphisms on inflammation, we quantified the activation of NF-κB during infection using the SEAP reporter assay (Fig. 8A). Compared to the mock control, infection with *H. pylori* 7.13 wt and Δ*cagL/cagL*^NK^ mutant displayed highly induced NF-κB activation. In contrast, the Δ*cagL* mutants exhibited strongly reduced NF-κB levels. Interestingly, infection with the Δ*cagL/cagL*^YE^ mutant induced intermediate NF-κB levels that significantly differed from infection with both the 7.13 wt and Δ*cagL* mutant (Fig. 8A). In addition, the supernatants from the same set of experiments were subjected to IL-8 chemokine ELISA, showing similar results (Fig. 8B). Together, these experiments indicate that 7.13 wt and Δ*cagL/cagL*^NK^ mutant progressively trigger NF-κB activation and IL-8 secretion, while the Δ*cagL/cagL*^YE^ point mutant was somewhat attenuated in these responses. As expected, the Δ*cagL* deletion mutant failed to induce these responses.

### CagL-dependent β-catenin activation

The Wnt-β-catenin signalling cascade dictates important tissue differentiation processes by controlling cell development, proliferation, and also cancer progression [82]. In healthy epithelial cells, β-catenin is a component of the E-cadherin-based adherens junction complex that maintains intercellular cell-to-cell adhesion. We have previously shown that cleavage of E-cadherin by *H. pylori* HtrA [20, 52] and T4SS-mediated injection of CagA [19] releases β-catenin from this complex, which subsequently translocated into the nucleus, followed by β-catenin*/*LEF-1-dependent cell proliferation. To investigate whether the CagL polymorphisms also play a role in β-catenin activation, we implemented TOP-/FOP-flash luciferase reporter assays. Similar to the CagA phosphorylation and cell scattering results described above, infection with *H. pylori* 7.13 wt and Δ*cagL/cagL*^NK^ mutant induced severe β-catenin activation compared to the mock control, while the Δ*cagL* and Δ*cagL/cagL*^YE^ mutants revealed almost no β-catenin activation (Fig. 8C). Taken together, these results show that the 7.13-wt and the Δ*cagL/cagL*^NK^ mutants induce significant β-catenin activation, while the Δ*cagL* deletion and Δ*cagL/cagL*^YE^ mutated strains were attenuated in this response.

### Effective chromosomal DNA damage

Previous studies provided evidence that *H. pylori* infection causes DNA double-strand breaks (DSBs) in the host’s genome, resulting in chromosome fragmentation and accumulating mutations that collectively increase the risk of GC development (reviewed by [2, 83]). Therefore, we isolated genomic DNA from AGS cells infected with the above *H. pylori* strains, followed by separation of the DNA using pulsed field gel electrophoresis (PFGE). Quantification results indicate that infection with *H. pylori* 7.13 wt and Δ*cagL/cagL*^NK^ mutant strains induced severe DNA fragmentation upon infection, whereas AGS cell infection with the Δ*cagL* and Δ*cagL/cagL*^YE^ mutants revealed less DNA damage (Fig. 8D, Supplementary Figure S5). However, similar to NF-κB activation and IL-8 secretion, infection with the Δ*cagL/cagL*^YE^ mutant induced an intermediate phenotype that differed from both 7.13 wt and Δ*cagL* mutant, suggesting a common mechanism in the pathogen-host interaction.

## Discussion

**Fig. 9:**
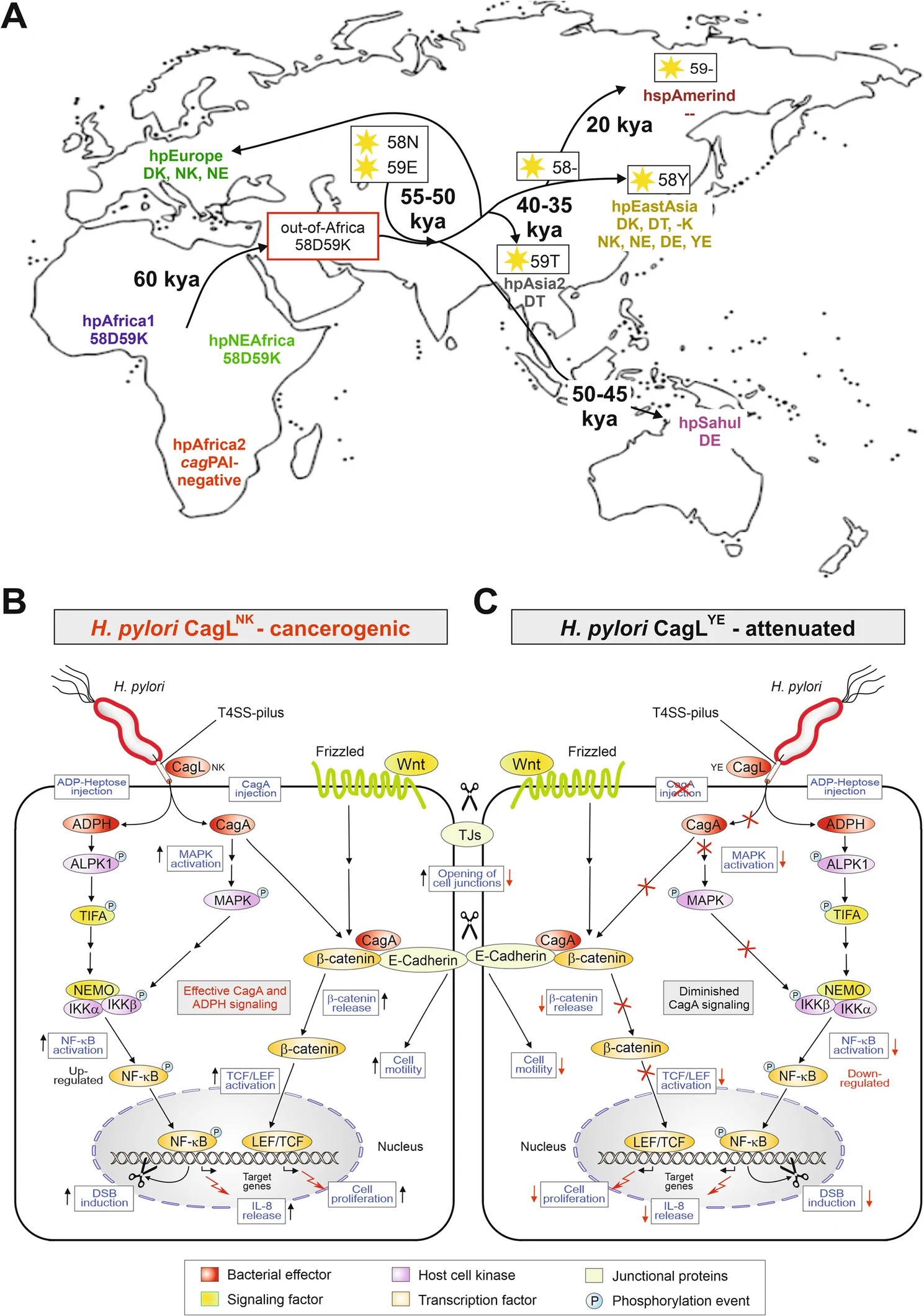
Evolution of *H. pylori*CagL polymorphisms in the hypervariable motifs during ancient human migrations out of Africa and models for cancer-associated signal transduction by NK- and YE-expressing strains. **A** Biogeographical distribution and evolution of hypervariable CagL motifs. The ancestral motif DK, found in the African CagL (hpAfrica1, hpNEAfrica), accompanied humans during their migration out of Africa about 60,000 years ago. Point mutations from D to N and from K to E, that likely occurred 55 − 50 kya, created variants DE that spread to Sahul and East Asia, and NK and NE present in strains from East Asia and Europe. Another point mutation around 35 kya generated allele DT found in hpAsia2 and hpEastAsia isolates. Subsequently, deletion mutations 58- and 59- appeared 35 − 20 kya, which generated variants -K and --, respectively, found in hpEastAsia and hspAmerind. Finally, allele 58Y arose through point mutation from N or D, which generated the YE variant present in East Asia. Colour code of the biogeographical populations as in Fig. 1B. **B** Model of GC-associated signalling induced by *H. pylori* 7.13 wt variant NK. The T4SS of *H. pylori* can translocate two major substrates, CagA and ADP-heptose (ADPH). Injected ADPH binds to and activates alpha kinase-1 (ALPK1). Active ALPK1 then phosphorylates the adapter protein TIFA. In turn, TIFA activates the downstream IKK signalling complex (composed of NEMO, IKKα and IKKβ) and finally p65 of transcription factor NF-κB, which is translocated into the nucleus to trigger IL-8 production. On the other hand, translocated CagA exhibits a supporting activity in this signalling by stimulating MAPK-mediated activation of Src kinase, which phosphorylates IKKβ (Y-199) to fully activate NF-κB [81]. *H. pylori*-induced NF-κB activation also results in replication stress and the occurrence of double strand break (DSB)-triggered DNA damage [86]. In addition, intracellular CagA activates the Wnt pathway and binds E-cadherin to release β-catenin from the complex. β-catenin then translocates into the nucleus to stimulate transcription factor LEF/TCF-mediated cell proliferation. In addition, disruption of cell-to-cell junctions and MAPK activation result in enhanced cell motility upon infection. **C** Infection with *H. pylori* 7.13 carrying the YE variant of CagL leads to attenuated signalling in gastric epithelial cells, which is mainly caused by the inhibition of T4SS-mediated CagA translocation and subsequent downregulation of all CagA-dependent signalling pathways described above. However, ADPH-dependent NF-κB activation and induction of DSBs remain detectable to some extent, which results in an attenuated disease phenotype in infected gerbils. Black arrows mark upregulated signalling pathways, while red crosses and red arrows indicate the blocked or downregulated pathways

The hypervariable motifs in CagL proteins of 1,735 *H. pylori* isolates of worldwide origin contained 16 different amino acid motifs (positions 58 and 59), eight of which were rare and only identified in very few isolates (Supplementary Table S1). The other eight amino acid motifs, namely DK, DT, DE, NK, NE, YE, -K, and a gap (--), were frequently found in *H. pylori* from one or several phylogeographic populations (Fig. 9A). Similarly, analysis of the five amino acid motifs (positions 58 to 62) showed eight major motifs among the isolates, plus the motif --IGK that appeared to be predominant in our very small hspAmerind population (Supplementary Table S2). Concurrent with the findings of Gorrell and colleagues [59], the biogeographic distribution of the CagL motifs showed that isolates from Africa solely contained DKMGE, suggesting this represents the ancestral allele (Supplementary Tables S1 and S2, Figs. 1B and 9A) that was present in *H. pylori* accompanying modern humans during the migration(s) out-of-Africa. Subsequently, the other variants arose in Asia through a series of independent as well as successive point mutations. Point mutations of codon 59 K to 59E (AAA to GAA) and 62E to 62 K (GAA to AAA) resulted in variant DEMGK that subsequently spread to Sahul, the ancient continent comprising modern New Guinea, Australia and Tasmania. Since archaeological evidence suggests that anatomically modern humans settled in Australia approximately 50,000 to 45,000 years ago (50–45 kya) [84], this mutation likely occurred 55–50 kya before the divergence between hpSahul and hpEastAsia (Fig. 9A). In Sahul, DE is present among *H. pylori* isolates from Papua New Guinea, whereas hpSahul strains from Australia do not possess the *cag*PAI. However, we noted that this observation is based on a limited number of available genomes (*n* = 17 in each case). Around the same time or perhaps shortly after, a point mutation from 58D (GAY) to 58 N (AAY) resulted in the CagL motifs NK and NE, both of which subsequently spread throughout Europe and East Asia. In South Asia, a point mutation from 59 K (AAR) to 59T (ACR) generated the motif DT that is frequently found among isolates of the populations hpAsia2 and hpEastAsia, suggesting that this mutation occurred before the divergence between the two populations, which likely occurred about 40 − 35 kya [85]. Subsequently, a deletion mutation (58-) in either DK or NK generated variant -K before the split of hspAmerind from hpEastAsia, followed by a further deletion mutation in hspAmerind isolates that created the gap (--) variant. Finally, either through mutation of AAY (N) or GAY (D) to TAY, 58Y appeared in East Asia, which gave rise to allele variant YE (Fig. 9A). While populations hpAfrica1, hpNEAfrica, hpAsia2 and hspAmerind contained only a single variant each, the greatest diversity was observed among the hpEastAsia isolates (Fig. 9A; Supplementary Table S1).

Previous publications reported the association of specific CagL motifs with GC development. Yeh and colleagues [54, 55, 87] claimed a significantly increased frequency of the YE motif among CagL from GC patients, whereas other authors showed an increased GC risk for the DK motif [56, 57] or the NE motif [58]. Unfortunately, these geographically restricted studies from Taiwan, India, Turkey and Iran were conducted with relatively small patient cohorts, which raises certain doubts about the statistical robustness across datasets. In addition, a study of CagL in 309 *H. pylori* isolates of worldwide origin found 58D underrepresented in samples from patients with GC [59], opposing the proposed GC association of DK reported from India and Turkey [56, 57]. Our present data, however, show that DK and NE are the predominant CagL alleles among hpEurope *H. pylori* isolates (Supplementary Table S1). HpEurope is characteristic for isolates from Turkey and Iran [4, 88] and also India [89], where additionally hpAsia2 (allele DT) is present. Thus, it is surprising that the GC association data from Iran (allele NE) contradict those from Turkey and India (allele DK). Allele DK is also the most prominent hypervariable motif of CagL in hpEastAsia isolates, whereas NE appears to play a minor role. Interestingly, the YE motif is only present among *H. pylori* from East Asia, and was reported to be overrepresented among a relatively small number of GC samples [54]. However, contrary to the above studies, we detected no GC correlation for any of the CagL motifs from 1,735 *H. pylori* strains, neither among the hpEurope isolates nor among the hpEastAsia isolates.

Besides its important role for the function of the *H. pylori* T4SS in CagA translocation and NF-κB-mediated IL-8 release [32, 38, 44], CagL exhibits several other tasks such as the control of integrin α5β1- and αvβ3-mediated cell elongation [38, 40, 90], α5β1-dependent cell spreading [91] and gastric acid production [92], αvβ5-facilitated gastrin expression [41] as well as TLR5 binding and activation [43]. These activities are primarily regulated by an RGD motif and a TLR5-binding sequence, both of which are located in close proximity to the hypervariable motifs in CagL (Fig. 1A). Crystallographic assessment of seven independent CagL structures derived from *H. pylori* strain 26695 revealed extensive physical plasticity within the protein [93]. In order to convert CagL into a more stable protein, intramolecular disulfide bridges were introduced genetically. This reduced CagL flexibility, but the capability for host cell adhesion was abrogated [93]. These findings led to the conclusion that CagL must partly unfold to promote host receptor engagement. In fact, CagL monomers form a bundle of four long α-helices (α1 to α4) in combination with two short α-helices (Fig. 1D [93])​. In addition to the described monomers, CagL was reported to form a domain-swapped dimer that was detected during the biochemical purification procedure [94]. In these molecules, the RGD-motif is positioned inside the α2-helix and is surface-exposed. Nevertheless, the adhesion of CagL to host target cells was firmly dependent on RGD presence [38, 91, 93]. The crystal structure of another CagL variant from the Asian strain K74 also displayed a bundle of helices as described for strain 26695, but two additional salt bridges were discovered in K74 between the helices, suggesting some more structural flexibility among natural CagL variants [95]. In both cases, the hypervariable motifs of CagL are found in a loop connecting the α1- and α2-helices (Fig. 1E). It is therefore tempting to speculate that mutational changes at amino acid positions 58/59 are under evolutionary pressure as they could permit changes in CagL structure, leading to modified receptor binding that could regulate T4SS-mediated transport of CagA and ADP-heptose.

We further investigated the role of the hypervariable CagL motifs NK and YE by in vivo and in vitro experiments using *H. pylori* strain 7.13, which has been previously shown to cause GC in the Mongolian gerbil animal model [19]. Our present results confirm that this wt strain (58N/59K) can robustly trigger typical GC-associated signal transduction processes in gastric epithelial cells, such as translocation of oncoprotein CagA into epithelial cells and the activation of MAP kinases ERK1/2, JNK and p38 —which are associated with the onset of nuclear responses and cell motility— as well as activation of the proliferation-associated β-catenin, proinflammatory transcription factor NF-κB-dependent IL-8 secretion and the induction of DNA damage through the introduction of DSBs in the host chromosome via published pathways discussed above and summarized in a model (Fig. 9B). In contrast, infection with 7.13 carrying the CagL 58Y/59E mutation exhibited a widely attenuated phenotype in these responses during infection of AGS cells (model in Fig. 9C). This is in agreement with our infection data in the Mongolian gerbil animal model system. While infection with wt strain 7.13 (58N/59K) and the *cagL* complemented strain (58​N/59K) triggered strong inflammatory responses and the development of adenocarcinoma in the animals, infection with the 58Y/59E mutant did not; in this case, only a slight increase in pathology was observed compared to the Δ*cagL* deletion mutant (Figs. 2 and 3). In fact, the YE strain phenotypically mimicked the attenuated clinical presentation of a Δ*cagA* deletion mutant in infected gerbils [96]. These observations were not attributable to differences in the colonization rates of the strains in gerbils (Fig. 2A) and AGS cell lines (Supplementary Figure S4) nor to differential expression or size changes of CagL, CagA and CagY (Fig. 4C/E, Supplementary Figure S3). Furthermore, using electron microscopy we observed pronounced T4SS-pilus formation by both complemented strains similar to 7.13 wt (Fig. 5), confirming that the T4SS machinery is basically expressed. We therefore assume that the YE mutant clones do not exhibit polar mutations, but were severely impaired with regard to CagA transport and the induction of CagA-dependent signalling pathways. However, NF-κB activation is mainly driven by two *H. pylori* factors, ADP-heptose and CagA [81]. Interestingly, the ADP-heptose-dependent portion of NF-κB activation and IL-8 secretion was preserved during infection of AGS cells, while the corresponding CagA portion was lost (Figs. 8A/B and Fig. 9C). These new findings are particularly noteworthy, because the formation of T4SS-pili by *H. pylori* clearly correlates with CagA translocation [38, 97, 98], but apparently is not sufficient on its own. Thus, our present study is the first showing that natural mutation in two codons of a structural T4SS protein (58Y/59E in CagL) still allows pilus formation and ADP-heptose mediated NF-κB activation and IL-8 secretion, but blocks CagA delivery and its downstream responses. This phenomenon is striking and should be investigated more closely in future experiments.

Previous in vitro infection studies by other groups have shown that *H. pylori* strains expressing YE in the hypervariable region of CagL, such as natural isolate 1033 [87], the isogenic *cagL*^YE^ mutant of gastritis strain 26695 or PUD strain P12 [61], induced substantial phosphorylation of CagA compared to control strains. The reason for this discrepancy is not clear. A possible explanation is that CagL mutations at positions 58/59 could interact with polymorphisms at other sites in CagL or with polymorphic sites in its interaction partners CagH and CagI [44] to regulate T4SS functions and gastric disease development. However, the YE mutation of CagL in our isogenic GC strain 7.13 resulted in a markedly attenuated phenotype in gastric disease development in the gerbils compared to infection with the wt NK polymorphism. Thus, the YE sequence does not predetermine that a given strain is cancerogenic. In fact, our data with strain 7.13 suggest the opposite, because GC development was blocked in both in vivo and in vitro experiments. Taken together, the data accumulated here and by various other studies suggest that the YE polymorphism may or may not affect CagA translocation, which was the actual rationale and starting point of our current study. The observed differences could be related to other polymorphisms in CagL or its interaction partners, which may occur specifically or more frequently in one or the other of the biogeographic *H. pylori* populations. While DKMGE represents the ancestral CagL allele from Africa and is the most common variant among hpEurope isolates, the variants YEMGE and DEMGE constructed by Tafreshi and colleagues [61] were not detected at all in the expanded dataset of natural *H. pylori* isolates (Supplementary Table S2). In addition, NEMGE from strains 26695 and P12 was extremely rare across the entire dataset as only isolates such as 26695 possessed this variant, and NKMGE was found in only four hpEurope isolates (2 gastritis and 2 PUD strains). In contrast, we have previously constructed a YEIGK variant [60] that is common in isolates from the hpEastAsia population, including isolates from patients with gastritis, gastric cancer and PUD (Supplementary Table S2). However, YE has evolved exclusively in East Asia, which is why this variant is not found in isolates from the hpEurope population, to which strain 26695 that was used in that study also belongs. Finally, the GC strain 7.13 used in the present paper carries the NKIQK motif that is the third most common motif among hpEurope strains and also occurs occasionally in hpEastAsia isolates (Supplementary Table S2). Accordingly, the YEIGQ mutant was constructed to match the 7.13 wt motif. Given that the previous studies reported that Y58E59 [54, 55] as well as E59, I60 and Q62 [59] are associated with GC, it was expected that the resulting YEIGQ mutant would be cancerogenic, but it was not. However, we note that YE only evolved in East Asia, and thus this variant is not present in hpEurope isolates to which strain 7.13 belongs. The motif YEIGQ occurred rarely in our expanded dataset and was found in only three isolates obtained from patients with PUD in China and Japan.

Interestingly, in addition to the hypervariable CagL motifs, several other genetic polymorphisms in *H. pylori* virulence factors have recently been identified and studied for their function. For example, an alanine/threonine polymorphism was detected in the EPIYA-B phosphorylation motif of CagA, which de-regulated β-catenin and NF-κB signalling pathways involved in GC development [51, 53]. Furthermore, a serine/leucine polymorphism in the serine protease HtrA was associated with trimer formation and increased proteolytic activity, which, in combination with other factors, could lead to malignant changes [52]. In both cases, the more aggressive alleles of HtrA and CagA appeared first during the evolution of *H. pylori*, while the less aggressive alleles emerged only later [99]. Thus, it turned out that these mutations in HtrA and CagA attenuated the virulence of the bacterium, making them prime examples of *H. pylori*’s elegant evolutionary adaptation to the human host. Similarly, we demonstrated here that the NK variant in CagL represents an aggressive, GC-associated allele, whereas the mutation to YE emerged later in Asia and appears to be an attenuated version of CagL, at least in the genetic background of the GC strain 7.13 (Fig. 9A). Moreover, previous studies using mouse and macaque animal models of *H. pylori* infection have shown that recombination events in specific repeat sequences of the T4SS core protein CagY is controlled by the host’s immune system to regulate the onset or termination of T4SS-mediated IL-8 production, probably by regulating T4SS-mediated injection of CagA and ADP-heptose [100]. GC-associated genetic variation has been also reported in the signal (s) and mid (m) regions of toxin VacA with the s1/m1 allele exhibiting the strongest cell damaging activity [23, 24, 50]. Overall, these results and our current findings support the hypothesis that the emergence of genetic polymorphisms in *H. pylori* virulence factors CagL, CagA, HtrA, VacA and CagY may act as molecular regulators that control T4SS-mediated host inflammatory responses, CagA translocation, and other cellular reactions. Future studies should examine in greater detail the role of such genetic polymorphisms during the course of *H. pylori* infection in patients with different gastric disease manifestations.

## Supplementary Information

Supplementary Material 1: Supplementary Figure S1. Cloning strategy and genetic complementation of H. pylori cagL. (A) CagL and flanking genes cagN and cagI from H. pylori strain 7.13 were amplified by PCR and cloned into the pGEM-Teasy vector (Promega Corporation, Madison, Wisconsin, USA) to generate plasmid pGEM-Teasy-7.13cagNLI. (B) The entire cagL gene from its start codon to the cagI stop codon was excised using the indicated primers and replaced by the kanamycin resistance gene (aphA3) to generate plasmid pGEM-Teasy-7.13 ∆cagL::aphA3 for transformation into 7.13. to create the ∆cagL deletion mutant. (C) Afterwards, we inserted a BglII restriction site in plasmid pGEM-Teasy-7.13cagNLI by the QuikChange site-directed mutagenesis (Agilent Technologies, La Jolla, California, USA) immediately after the cagL stop codon using the indicated primers. This site was used to insert the chloramphenicol resistance cassette (cat) with compatible BamHI sites to generate plasmid pGEM-Teasy-7.13 ∆cagLNK wt::cat. (D) pGEM-Teasy-7.13 ∆cagLNK wt::cat was further modified to introduce N58Y and K59E mutations using the indicated primers and QuikChange kit. The resulting complementation plasmid pGEM-Teasy-7.13 ∆cagLYE::cat as well as pGEM-Teasy-7.13 ∆cagLNK wt::cat were transformed into the 7.13 ∆cagL deletion mutant to restore CagL expression by the NK wild-type (wt) or YE alleles, respectively. Blue arrows indicate the position and names of primers. All primers are listed in Supplementary Table S3. All clones were checked by restriction digests and sequencing. Supplementary Figure S2. Cross-structure statistics. The RMSD matrix indicates the pairwise similarity between any two CagL models generated in this study. The lower the value, the more similar the models are. Structures 1–5 all have RMSD values of < 0.8 Å relative to each other. Model 6 with the YE motif shows RMSD values > 1.1 Å relative to all other models. The largest structural difference (1.44 Å) is present between the models with the NK and the YE motif. Supplementary Figure S3. Western blot analysis of protein expression in isogenic H. pylori cagL mutant strains. All indicated bacteria were grown for two days on GC agar plates and protein lysates were prepared. Four individual clones each of our complemented CagL NK (wild-type, wt) and YE strains were compared to parental 7.13 wt and ∆cagL deletion mutant. No differences were seen among the strains regarding the expression of T4SS proteins CagL, CagA and CagY with urease B (UreB) as loading control, except for the lack of the CagL band in the ∆cagL deletion variant as expected. Supplementary Figure S4. Binding assays confirm similar attachment capabilities of H. pylori strains to AGS cells and E-cadherin-expressing AGS cells. (A) Cultured AGS gastric epithelial cells and (B) AGS cells that were stably transduced with wt human E-cadherin were cultured to about 80% confluency, followed by infection with the indicated H. pylori strains for 6 hours at an MOI of 50. Both cell lines revealed similar H. pylori binding capacities upon infection. About 40 CFU of bound bacteria per cell were detected, demonstrating similar cell binding of H. pylori to both cell types. Each point represents data from an individual experiment. All graphs display mean values ± SD derived from three independent biological replicates. No significant (ns) statistical differences were observed among the strains. Supplementary Figure S5. Representative PFGE gel from the experiments shown in Fig. 8D. Supplementary Figure S6. All uncropped Western blot files of Fig. 4, Fig. 7 and Supplementary Figure S3.

Supplementary Material 2.

Supplementary Material 3.

Supplementary Material 4.

## Data Availability

The datasets supporting the conclusions of this article are included within the article and its additional files. Materials are available upon request.
