# Supplementary material for: Evolution and function of genetic polymorphisms in the Helicobacter pylori type IV secretion pilus protein CagL in gastric carcinogenesis
Source: Cell Commun Signal. 2026 Oct 30;24:589. doi: 10.1186/s12964-026-03334-9 (PMC13721428; doi:10.1186/s12964-026-03334-9)
Supplement: Supplementary file 1 [file 12964_2026_3334_MOESM1_ESM.pdf]

# Figure S1

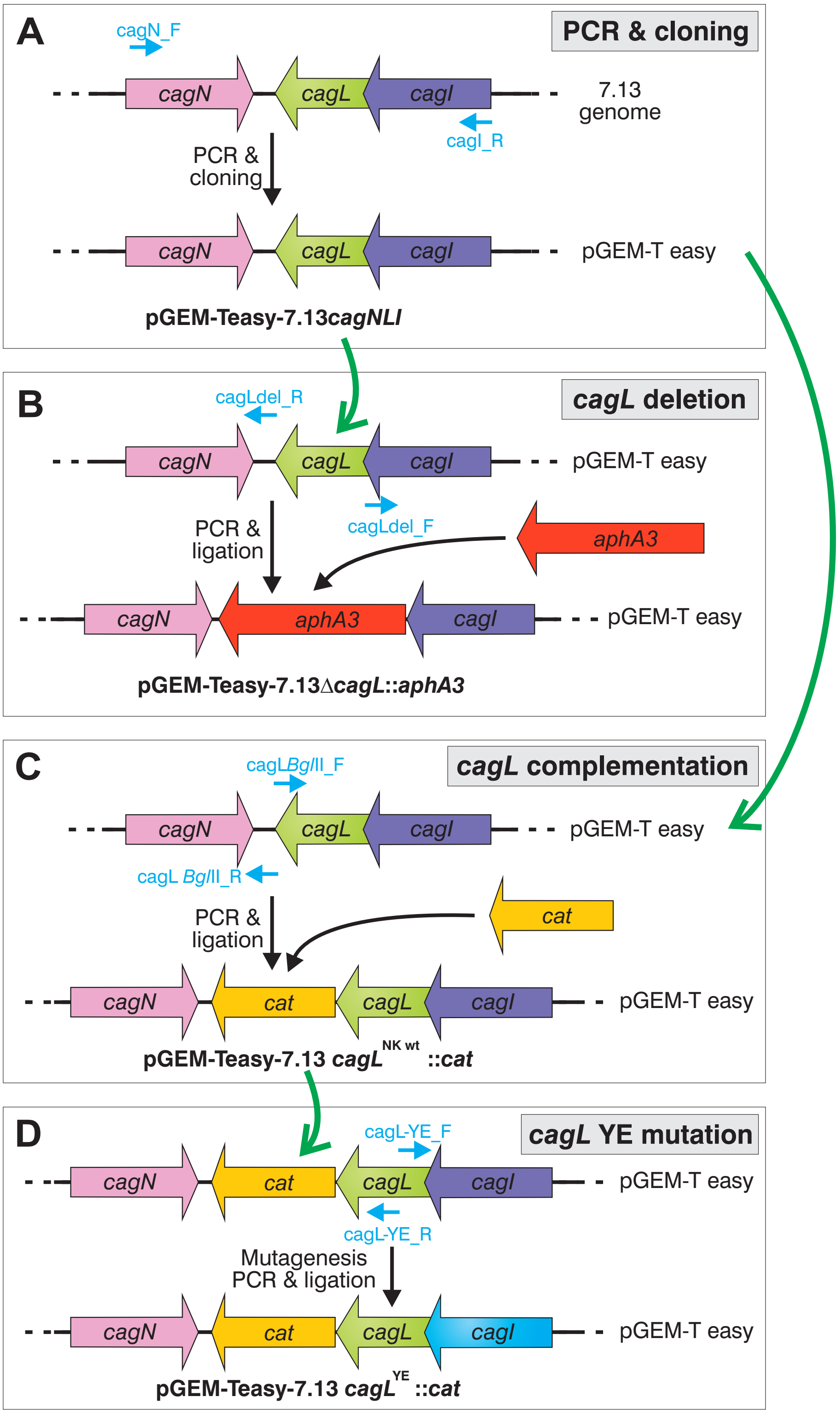

A
PCR & cloning
cagN_F
cagN
cagL
cagI
7.13
genome
cagI_R
PCR &
cloning
pGEM-T easy
pGEM-Teasy-7.13cagNLI
B
cagL deletion
cagLdel_R
pGEM-T easy
cagLdel_F
PCR &
ligation
aphA3
pGEM-Teasy-7.13ΔcagL::aphA3
C
cagL complementation
cagLBglII_F
cagL BglII_R
cat
pGEM-Teasy-7.13 cagL NK wt ::cat
D
cagL YE mutation
cagL-YE_F
cagL-YE_R
Mutagenesis
PCR & ligation
pGEM-Teasy-7.13 cagL YE ::cat

# Figure S2

## Cross-structure statistics.

### RMSD

| | structure: | 1 | 2 | 3 | 4 | 5 | 6 |
|---|---|---|---|---|---|---|---|
| 1 | cagl_dk_model_0.pdb:A | | 0.451 | 0.586 | 0.457 | 0.765 | 1.258 |
| 2 | cagl_de_model_0.pdb:A | 0.451 | | 0.450 | 0.362 | 0.541 | 1.310 |
| 3 | cagl_dt_model_0.pdb:A | 0.586 | 0.450 | | 0.581 | 0.756 | 1.434 |
| 4 | cagl_ne_model_0.pdb:A | 0.457 | 0.362 | 0.581 | | 0.651 | 1.166 |
| 5 | cagl_nk_model_0.pdb:A | 0.765 | 0.541 | 0.756 | 0.651 | | 1.440 |
| 6 | cagl_ye_model_0.pdb:A | 1.258 | 1.310 | 1.434 | 1.166 | 1.440 | |

# Figure S3

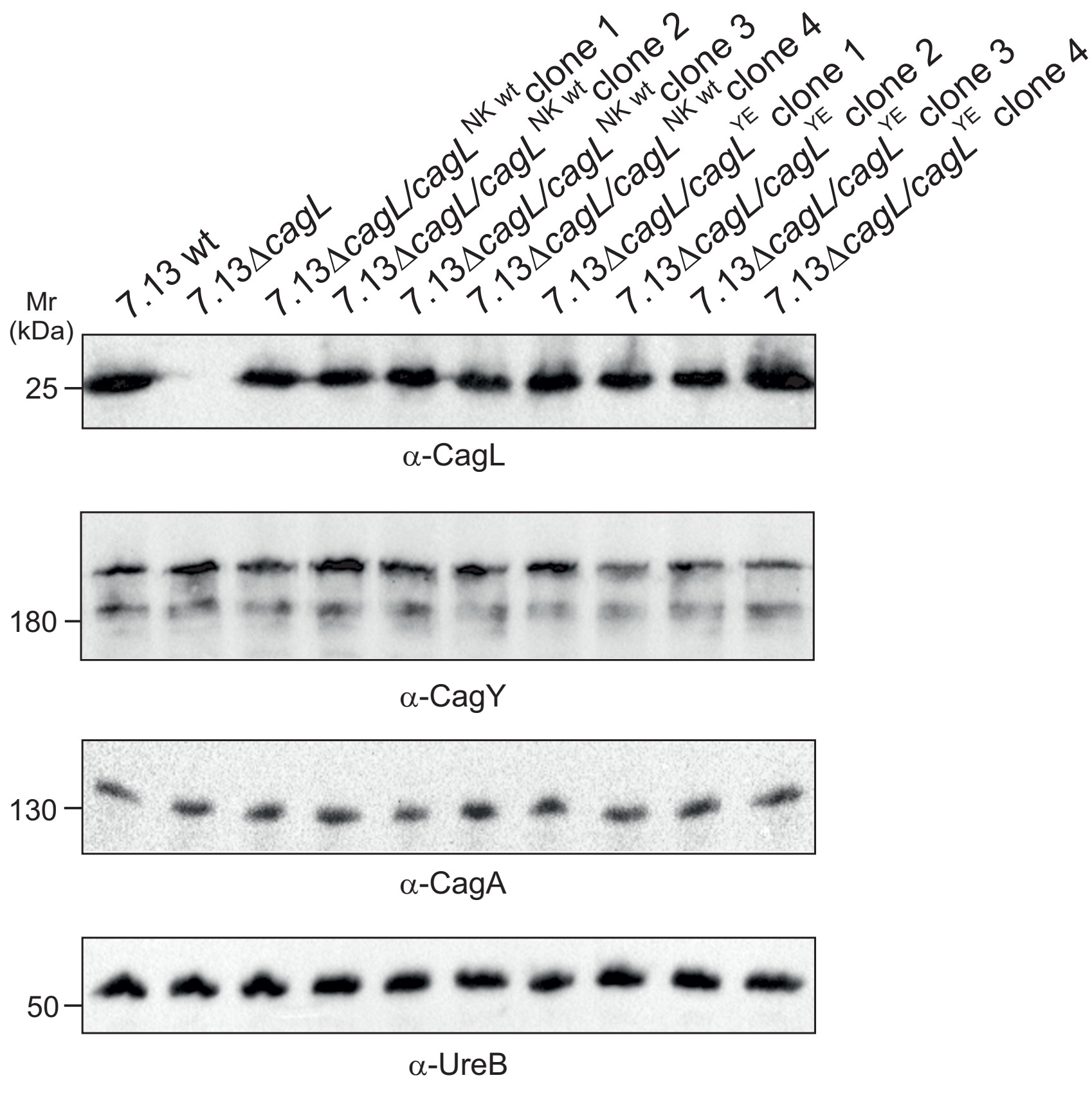

7.13 wt
7.13ΔcagL
7.13ΔcagL/cagL NK wt clone 1
7.13ΔcagL/cagL NK wt clone 2
7.13ΔcagL/cagL NK wt clone 3
7.13ΔcagL/cagL NK wt clone 4
7.13ΔcagL/cagL YE clone 1
7.13ΔcagL/cagL YE clone 2
7.13ΔcagL/cagL YE clone 3
7.13ΔcagL/cagL YE clone 4
Mr (kDa)
25
α-CagL
180
α-CagY
130
α-CagA
50
α-UreB

# Figure S4

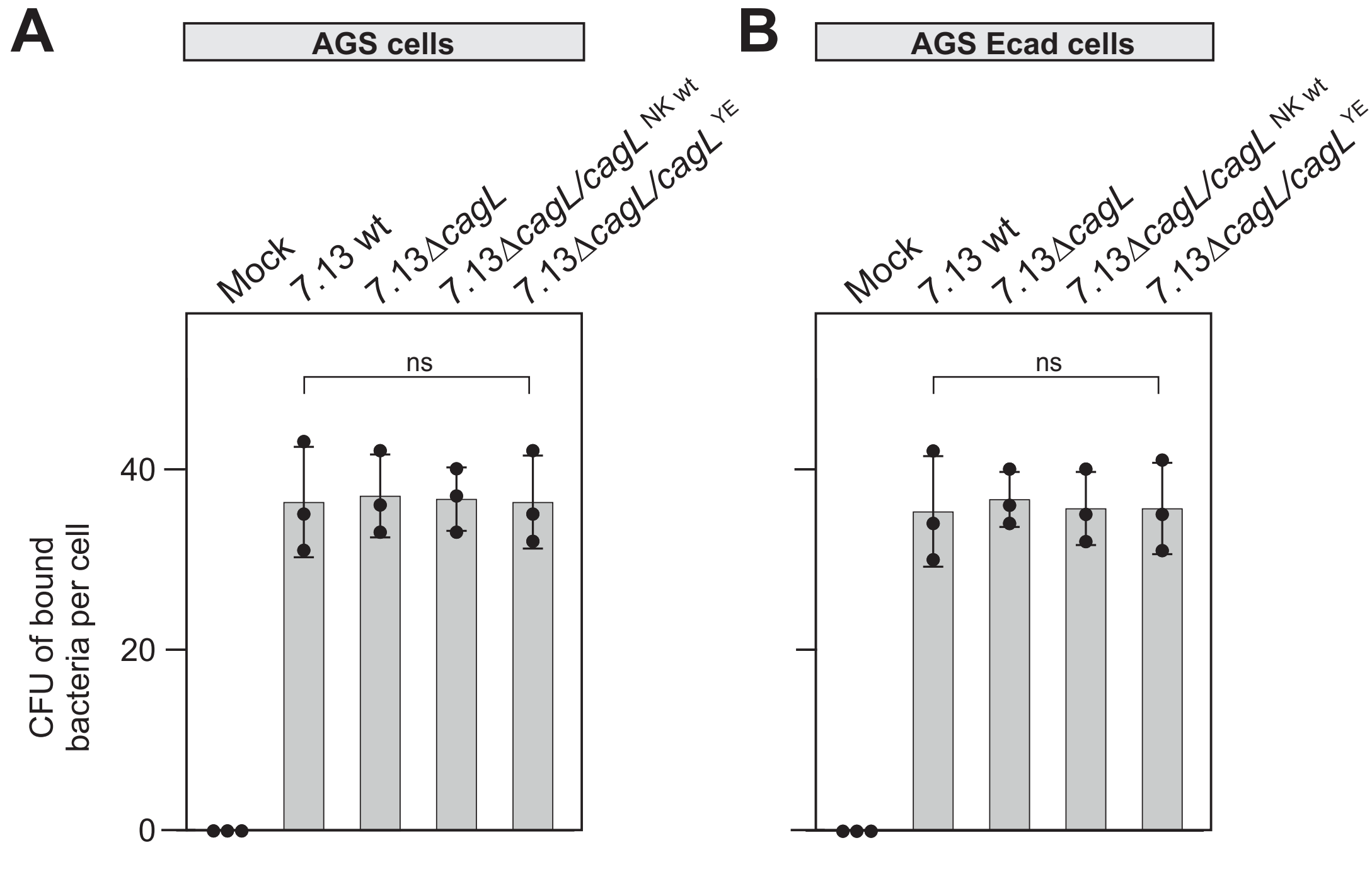

A
AGS cells
B
AGS Ecad cells
Mock
7.13 wt
7.13ΔcagL
7.13ΔcagL/cagL NK wt
7.13ΔcagL/cagL YE
ns
CFU of bound bacteria per cell
0
20
40

# Figure S5

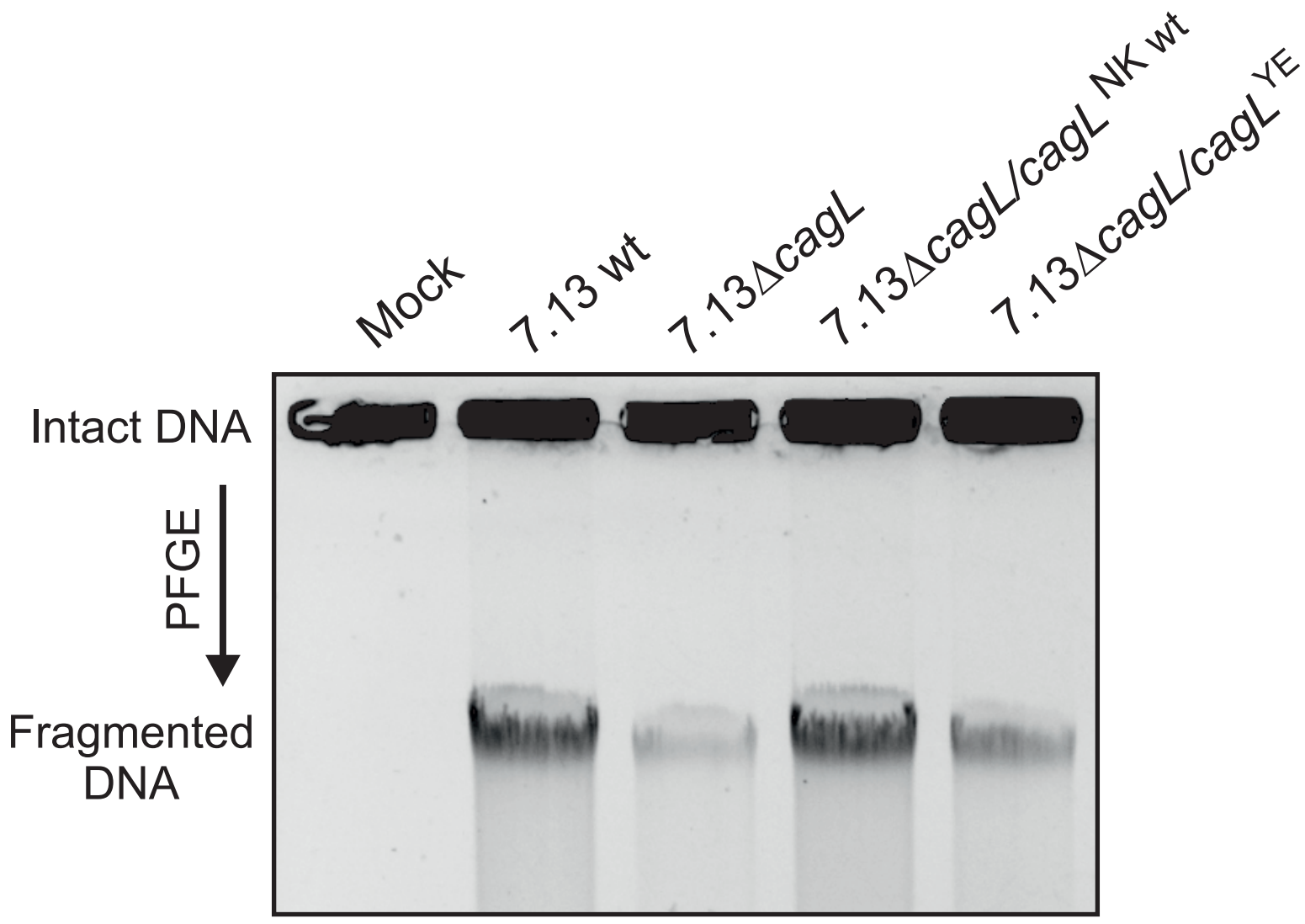
Mock
7.13 wt
7.13ΔcagL
7.13ΔcagL/cagL NK wt
7.13ΔcagL/cagL YE
Intact DNA
PFGE
Fragmented DNA

# Figure S6

## Uncropped blot files
### of Figure 4C

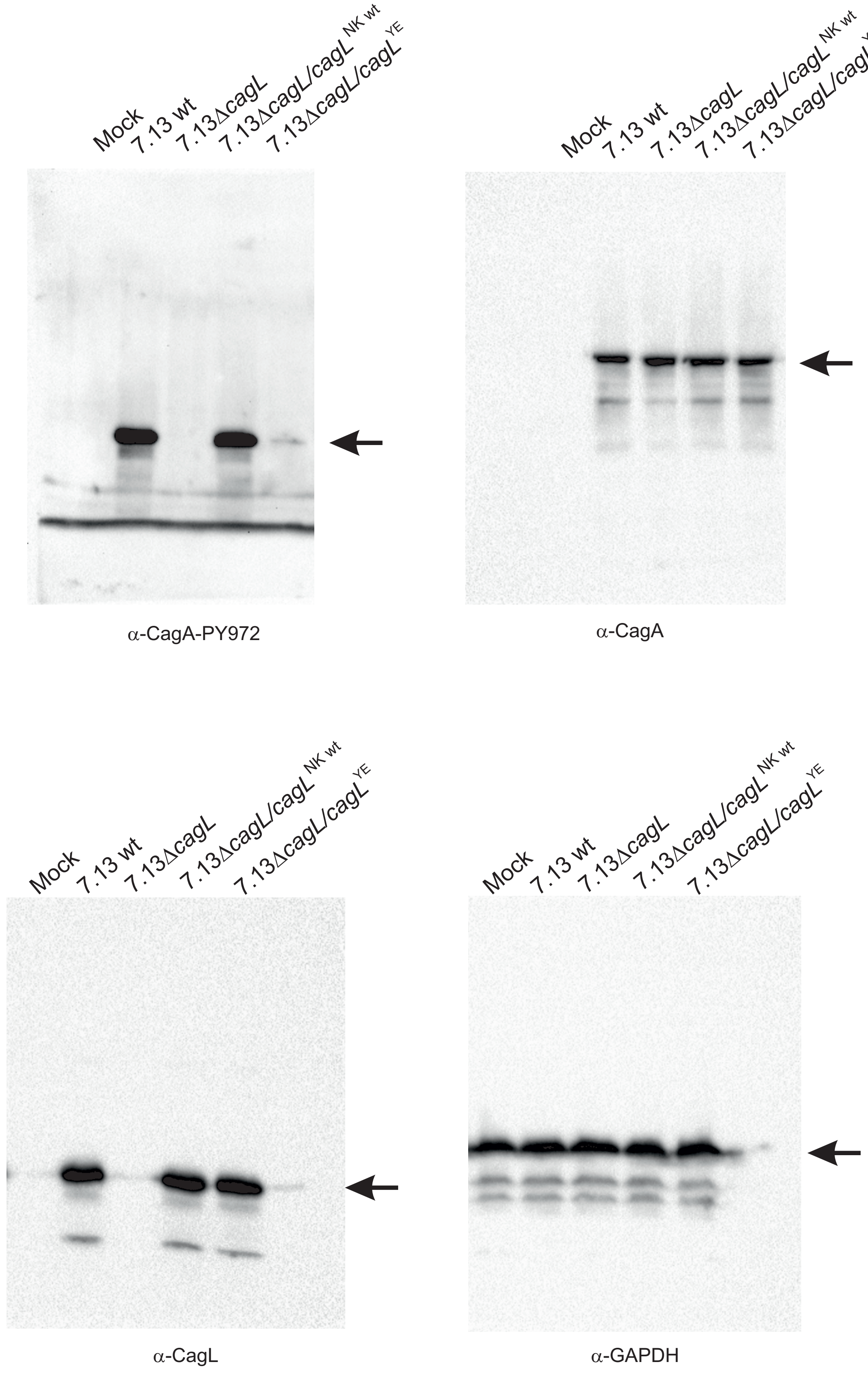

# Figure S6 continued

## Uncropped blot files
of Figure 7A

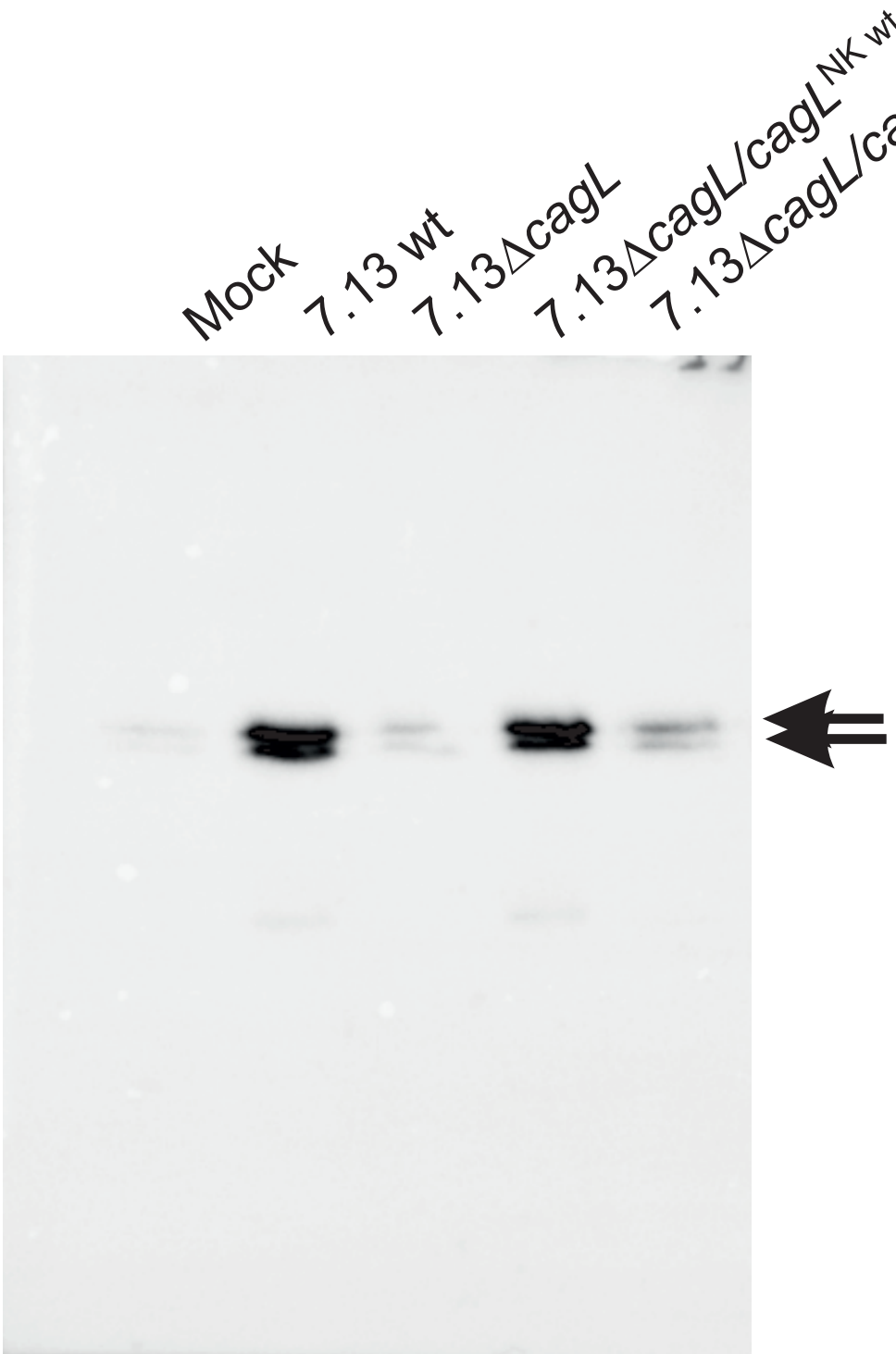


α-phospho-Erk (T202/ Y204)

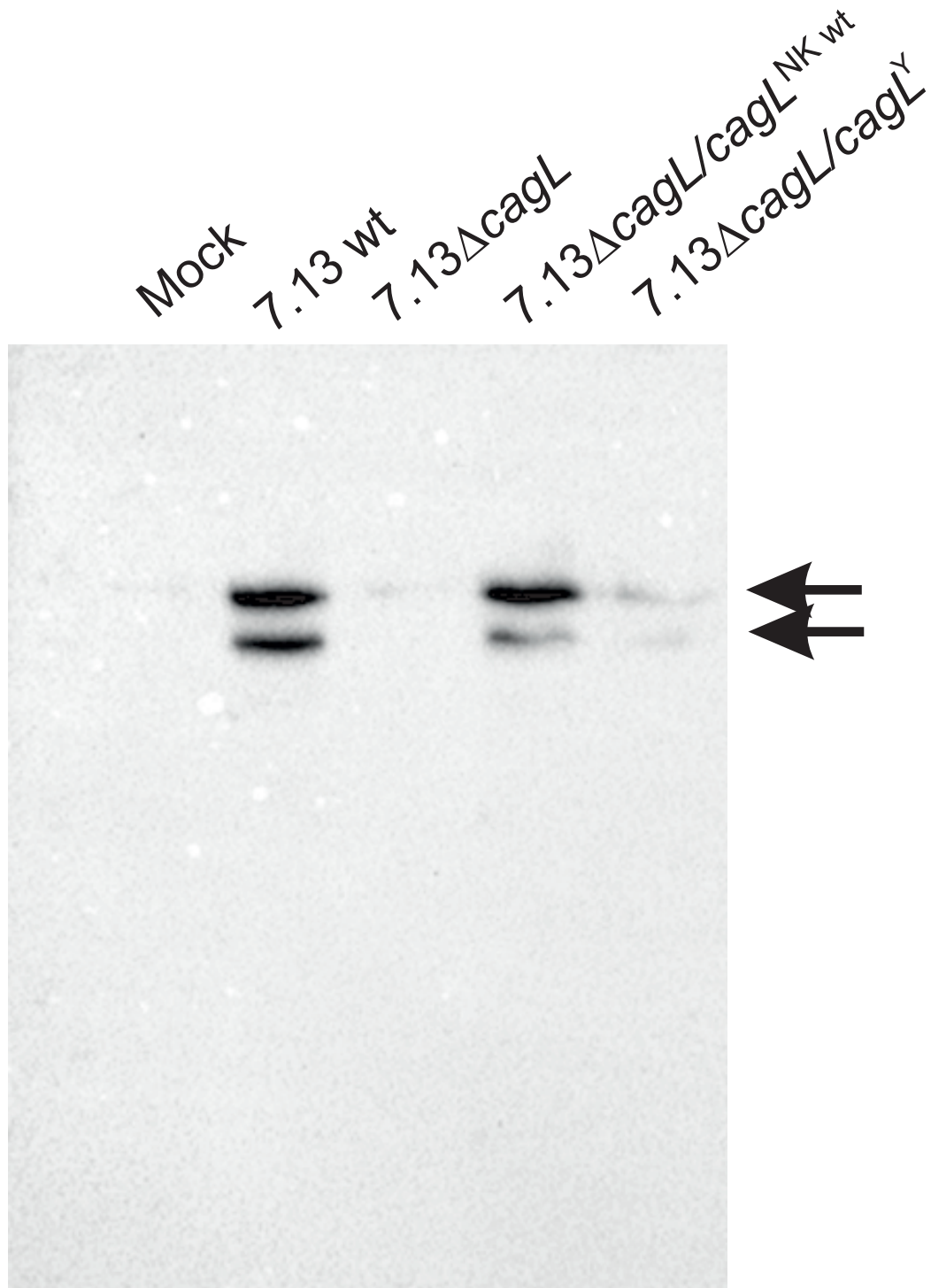


α-phospho-JNK (pT183/pY185)

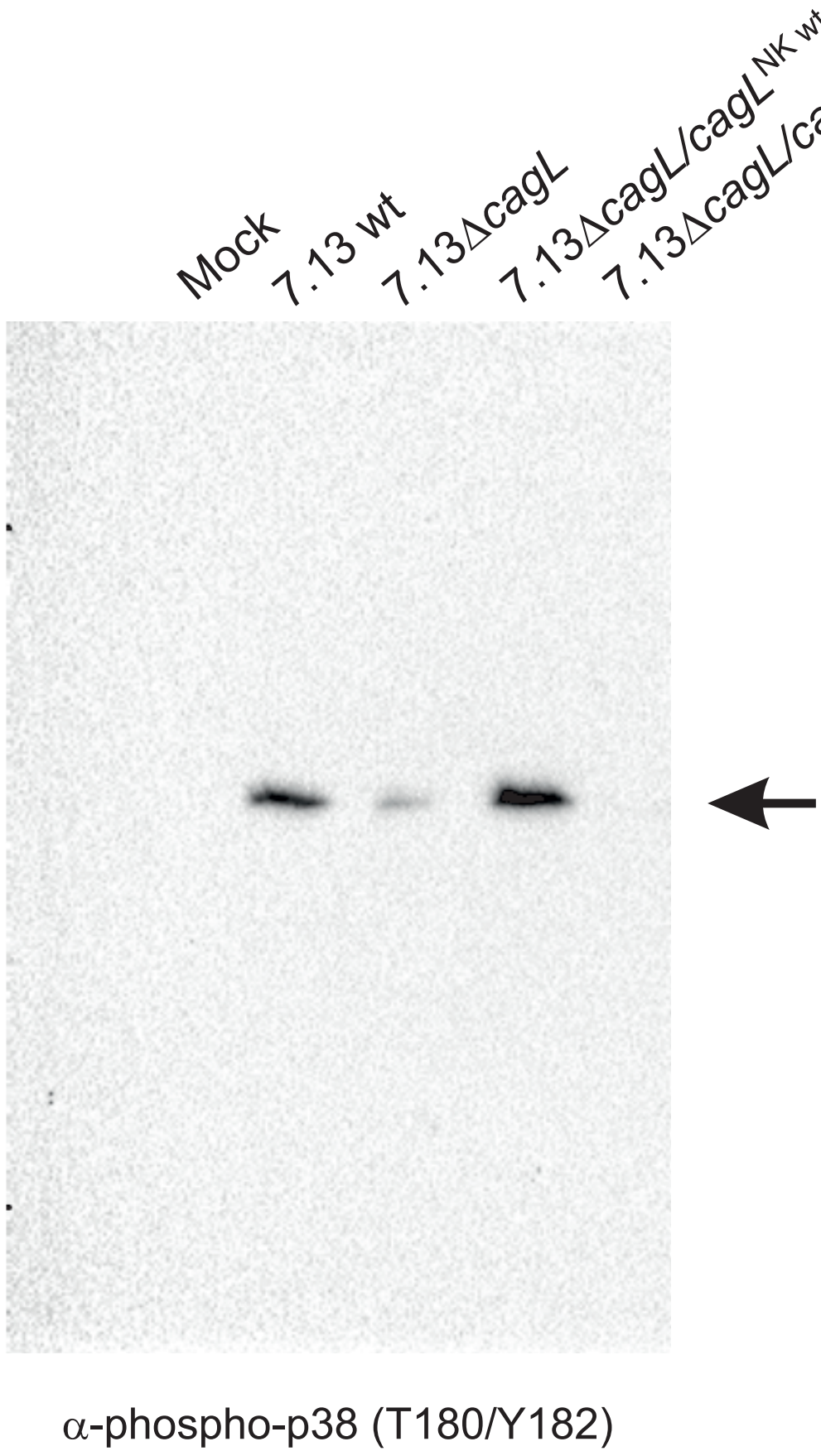


α-phospho-p38 (T180/Y182)

# Figure S6 continued

## Uncropped blot files
of Figure S3

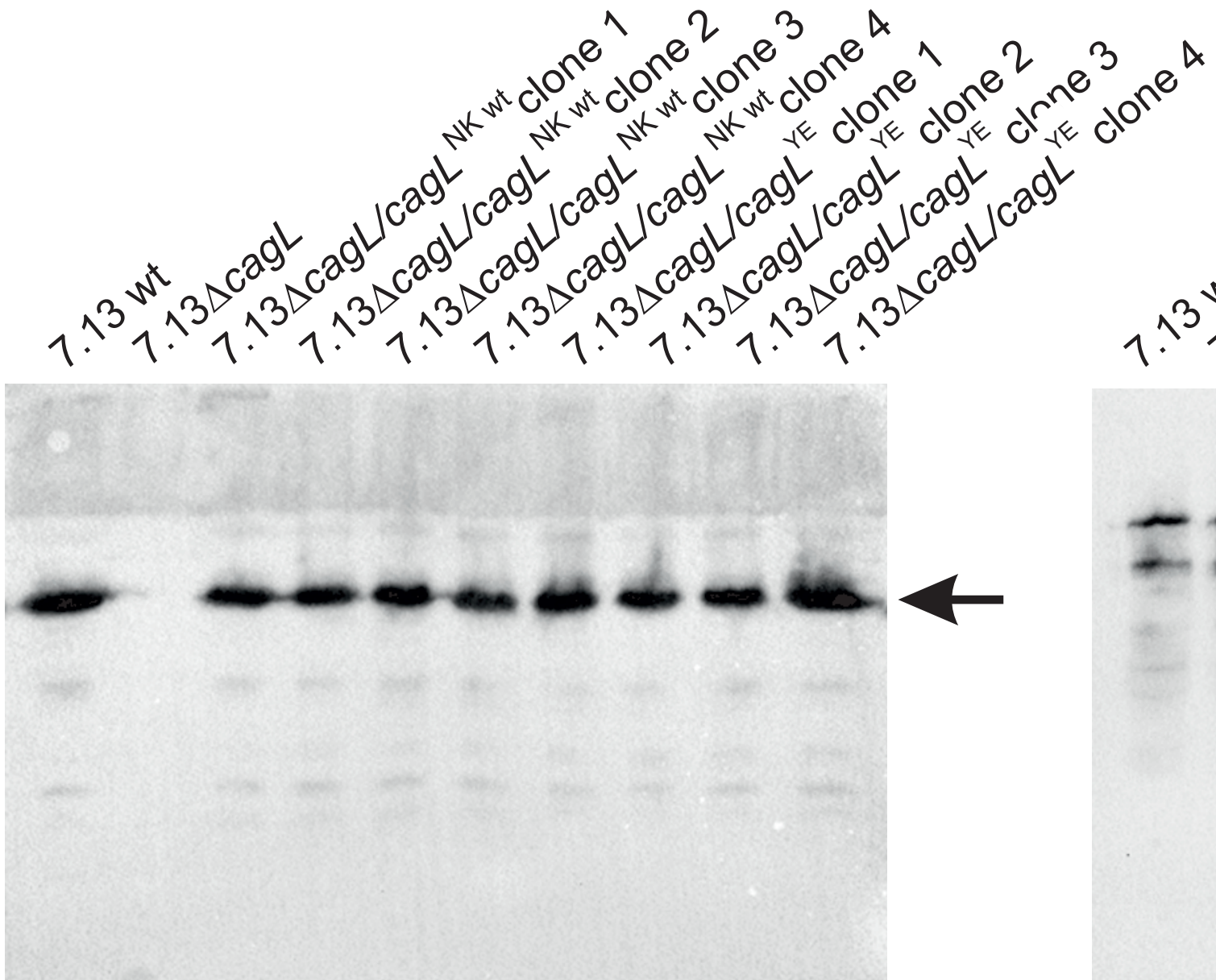

α-CagL

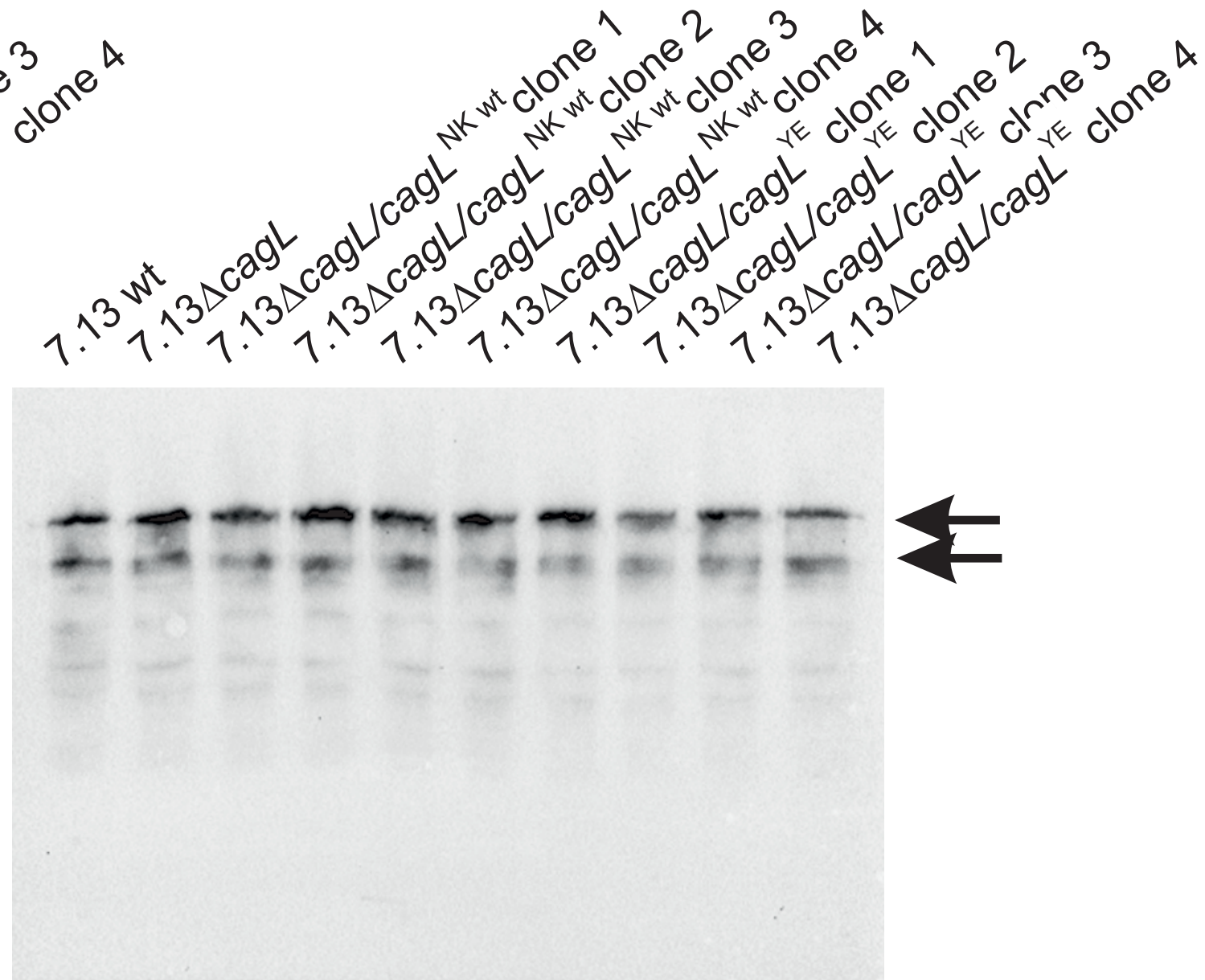

α-CagY

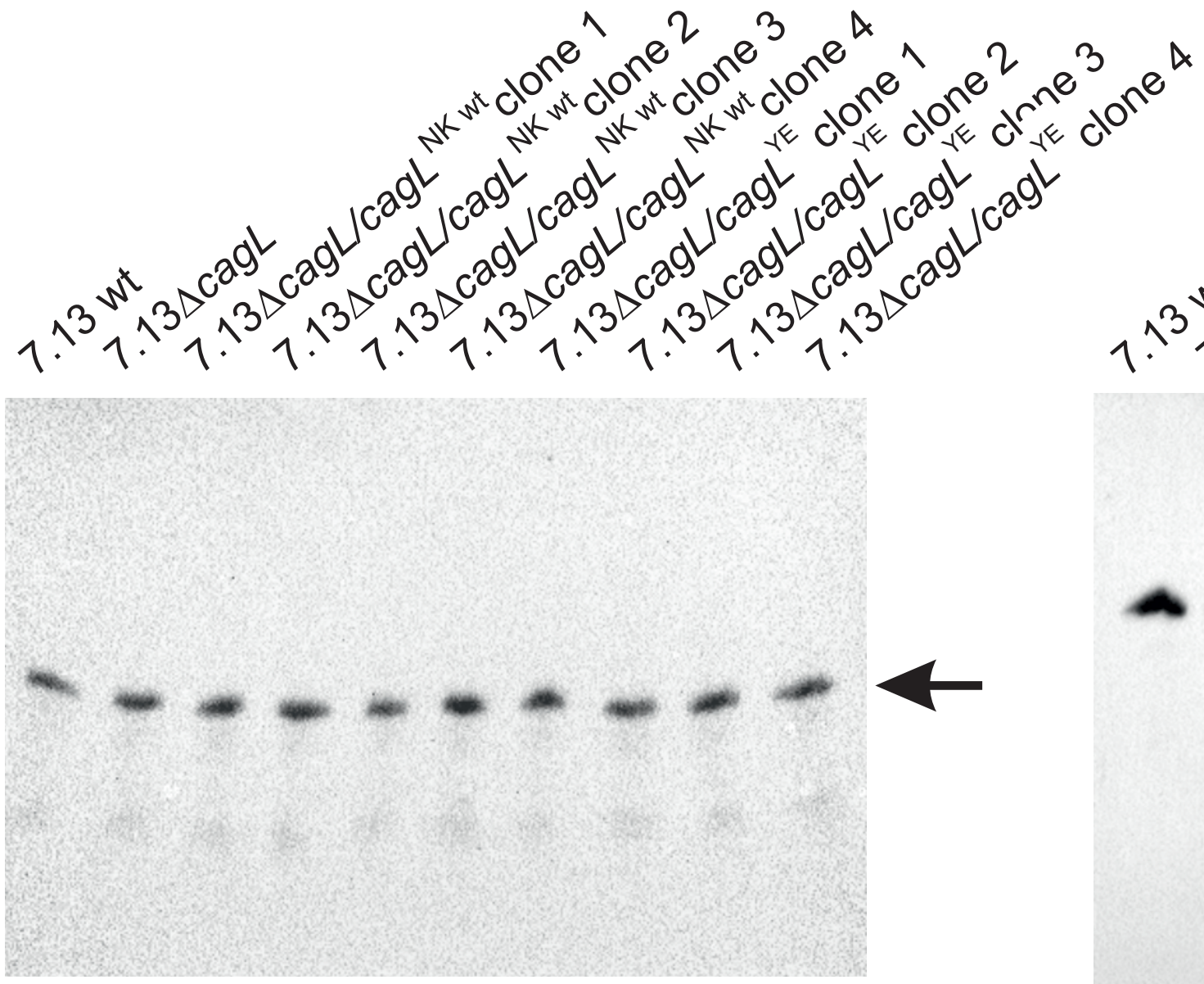

α-CagA

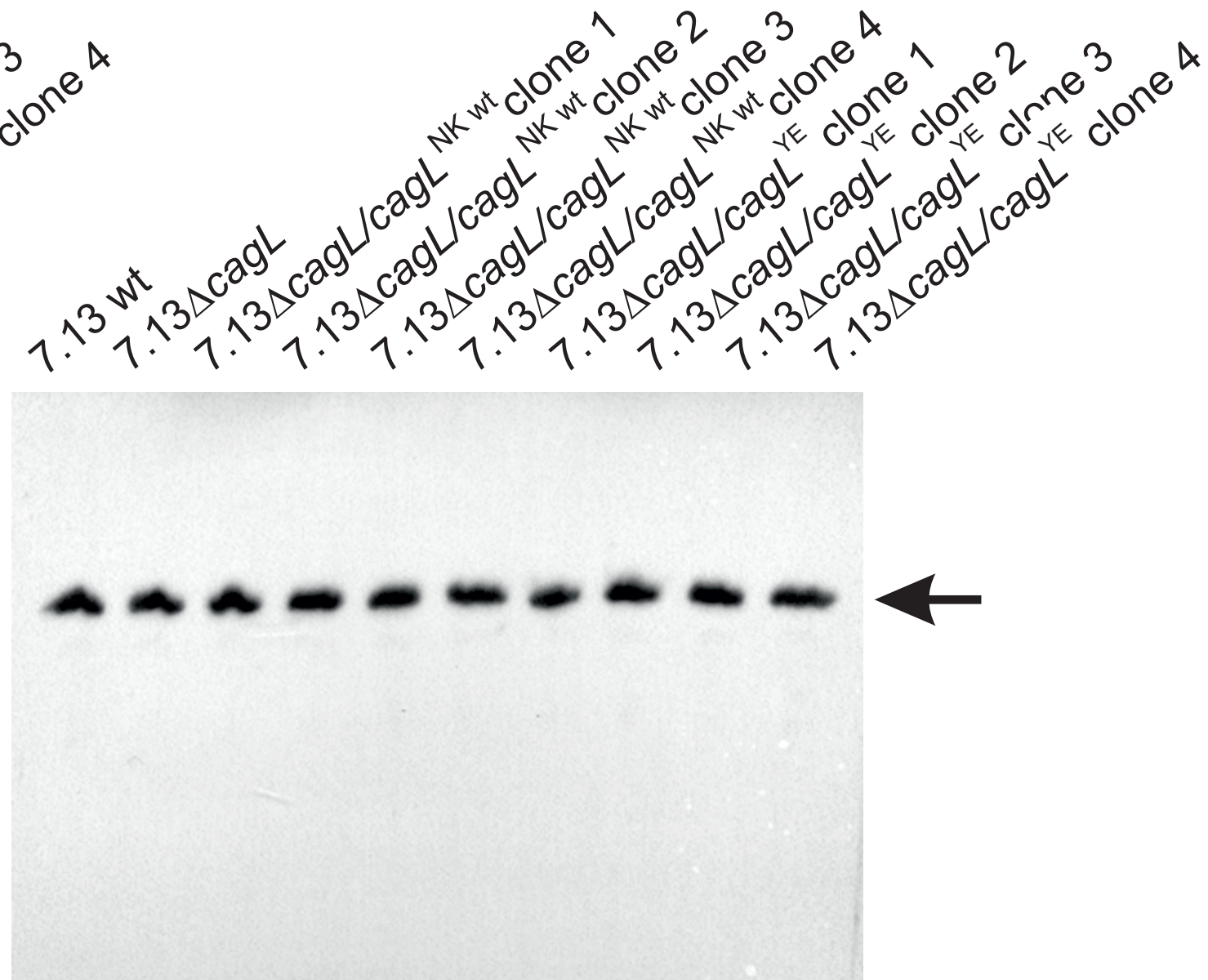

α-UreB
