# Supplementary material for: Evolution and function of genetic polymorphisms in the Helicobacter pylori type IV secretion pilus protein CagL in gastric carcinogenesis
Source: Cell Commun Signal. 2026 Oct 30;24:589. doi: 10.1186/s12964-026-03334-9 (PMC13721428; doi:10.1186/s12964-026-03334-9)
Supplement: Supplementary file 2 [file 12964_2026_3334_MOESM2_ESM.pdf]

Supplementary Table 1. Disease associations for CagL amino acid polymorphisms in 1,735 *H. pylori* isolates

**CagL Sequence**	**Number of isolates**																											
	hpEastAsia (East Asian countries)							hpAsia2 (Asian countries)							hpEurope (Non-Asian countries)							hpAfrica1 (Non-Asian countries)						
	Mild	AG	GC	MALT	Meta	PUD	**Total**	Mild	AG	GC	MALT	Meta	PUD	**Total**	Mild	AG	GC	MALT	Meta	PUD	**Total**	Mild	AG	GC	MALT	Meta	PUD	**Total**
DE	45	3	29	1	6	27	**111**							**0**														**0**
DI								1						**1**														**0**
DK	85	32	72	5	1	91	**286**	4						**4**	182	12	45	4	58	17	**318**	70	2	10	1	27	15	**125**
DN	2		1			2	**5**							**0**														**0**
DQ	1						**1**							**0**												1		**1**
DR	1	2					**3**							**0**														**0**
DT	28	26	24	1	1	47	**127**	65		4			10	**79**	1						**1**	2						**2**
GT		2	1			4	**7**							**0**														**0**
-K	15	4	4			8	**31**							**0**	1		2			2	**5**							**0**
NE	19	9	11	1		13	**53**	4						**4**	156	15	32	1	37	23	**264**							**0**
NG	1						**1**							**0**	1						**1**							**0**
NK	10	5	8	1		7	**31**						2	**2**	104	7	20	2	29	18	**180**	1					2	**3**
NT			2				**2**	1						**1**														**0**
YE	17	16	16	1	1	24	**75**							**0**														**0**
YG	2						**2**							**0**														**0**
**Total**	**226**	**99**	**168**	**10**	**9**	**223**	**735**	**75**	**0**	**4**	**0**	**0**	**12**	**91**	**445**	**34**	**99**	**7**	**124**	**60**	**769**	**73**	**2**	**10**	**1**	**28**	**17**	**131**

In addition: hpNEAfrica (Northeast Africa) 4 DK (4 Mild); hspAmerind (Indigenous Americans) 1 DK (Mild), 4 -- (3 Mild, 1 GC)

Mild - Normal mucosa and Non-atrophic Gastritis; AG - Atrophic Gastritis; GC - Gastric Cancer; Meta - Metaplasia; PUD - Peptic Ulcer Disease
