# Supplementary material for: Evolution and function of genetic polymorphisms in the Helicobacter pylori type IV secretion pilus protein CagL in gastric carcinogenesis
Source: Cell Commun Signal. 2026 Oct 30;24:589. doi: 10.1186/s12964-026-03334-9 (PMC13721428; doi:10.1186/s12964-026-03334-9)
Supplement: Supplementary file 3 [file 12964_2026_3334_MOESM4_ESM.pdf]

Table S3. PCR and DNA sequencing primers used in this study

Primer name	Primer sequence (5' → 3')
cagN_F	CAATCGCTCTAAATCTGAACTGCCC
cagI_R	CATGCCTTATGGAACAGGTTATTCAG
cagLdel_F	CGGGATCCTCATTTGACAATAACTTTAGAG[a]
cagLdel_R	CGGGATCCTTTTTCTGAGACGATAGGCTATG[a]
cagL*Bgl*II_F	GGTTAGAAATCATAGCCTATCGTCTCAGA**TCT**<br>ATCATTTAACAATGATCTTACTTGATTGC[b]
cagL*Bgl*II_R	GCAATCAAGTAAGATCATTGTTAAATGATAGA<br>**TCT**GAGACGATAGGCTATGATTTCTAACC[b]
cagL-YE_F	**G**AAATAGGTCAAGAAGATGCTCTAA[c]
cagL-YE_R	TT**C**ATAATTAGCGCTAGGGCTAGTG[c]
T7seq[d]	TAATACGACTCACTATAGGG
SP6seq[d]	CATTTAGGTGACACTATAG
cagLseq1[e]	CGCTTACTTTGTTCTAGGG
cagLseq2[e]	ACCACTAGCCCTAGTGC

a. engineered *Bam*HI site underlined
b. engineered *Bgl*II site underlined; modified nucleotides bolded
c. modified nucleotides bolded
d. Primers flanking the multicloning sites in pGEM-Teasy (to sequence inserts)
e. Primers to sequence *cagL* and flanking areas
